# Treatment of Rheumatoid Arthritis Using Combination of Methotrexate and Tripterygium Glycosides Tablets—A Quantitative Plasma Pharmacochemical and Pseudotargeted Metabolomic Approach

**DOI:** 10.3389/fphar.2018.01051

**Published:** 2018-10-09

**Authors:** Menglei Wang, Jing Huang, Huizhen Fan, Dan He, Siyu Zhao, Yisong Shu, Hui Li, Linlin Liu, Shuang Lu, Cheng Xiao, Yuanyan Liu

**Affiliations:** ^1^Department of Chinese Medicine Chemistry, School of Chinese Materia Medica, Beijing University of Chinese Medicine, Beijing, China; ^2^Institute of Clinical Medicine, China-Japan Friendship Hospital, Beijing, China; ^3^Jianguomen Community Health Service Center of Dongcheng District, Beijing, China; ^4^Department of Gastroenterology, People's Hospital of Yichun, Jiangxi Yichun, China

**Keywords:** rheumatoid arthritis, methotrexate, tripterygium glycosides tablets, combination treatment, quantitative plasma pharmacochemistry, pseudotargeted metabolomics

## Abstract

Rheumatoid arthritis (RA) is a chronic inflammatory disease characterized by chronic destructive synovitis and is associated with progressive disability, systemic difficulties, premature death, and socioeconomic costs. Early intervention with disease-modifying antirheumatic drugs (DMARDs) like methotrexate (MTX) and its combination regimen would provide obvious benefits to patients, healthcare systems and society. MTX and tripterygium glycosides tablets (TGT_S_) are most frequently prescribed medicines for RA, and the combination of them occurs frequently in anti-RA prescriptions. While the underlying combination mechanisms and the affected variation of drug blood level remain unclear. According to the American College of Rheumatology criteria for improvement, clinical evaluation following three treatment groups (i.e., MTX and TGT_S_ mono- and combined groups) were carried out at baseline and at the end of 12 weeks in a randomized controlled clinical trial. To monitor the affected variation of drug blood level and perturbation of metabolites caused by MTX plus TGT_S_ combined to treat active RA, the collected plasma samples were analyzed using RRLC-QqQ-MS and UHPLC-QE Orbitrap HRMS instruments. As a result, 39 metabolites including 7 MTX-related metabolites, 13 TGT_S_-related migratory ingredients and 19 characteristic endogenous metabolites, were quantitatively determined in plasma samples of RA patients after oral administration. The potential mechanism of MTX and TGT_S_ combination were preliminarily elucidated on the aspect of clinical biochemical test indicators integrated with quantitative plasma pharmacochemistry and the pseudotargeted metabolomics.

## Introduction

Rheumatoid arthritis (RA) is a chronic and systemic autoimmune inflammatory disorder that affects about 0.5–1.0% of total adult population and brings heavy burden on healthcare systems (Zampeli et al., 2015). RA is characterized by systemic and synovial inflammation, which leads to permanent joint damage and disability if uncontrolled. Disease-modifying antirheumatic drugs (DMARDs) are recommended as the first-line treatment for RA early clinical intervention. In global, methotrexate (MTX) as a DMARDs, owing to its efficacy, acceptable, and low cost, has become (and remains) a mainstay in the treatment of RA. As shown in recent estimates, MTX ranked as number 1st medicine in early RA occurs in over 90% of patients (Sokka and Pincus, 2008). MTX can enter variety of cells including RBCs, WBCs, hepatocytes, and synoviocytes through reduced folate carrier 1 or SLC19A1 after oral. Once inside the cell, MTX is converted to polyglutamates (MTXPGn) in the action of folylpolyglutamate synthase (FPGS) by sequential addition of glutamate residues (Ghodkepuranik et al., 2015). The MTXPGn ranged from MTXPG2 to MTXPG5 is mainly deposited intra-cellular. Increasing researches suggest that the therapeutic effects of MTX are attributed to the intracellular levels of MTXPGn (Den et al., 2013). Nevertheless, the mainly extracellular metabolites of MTX, including 7-hydroxy methotrexate (7-OH MTX) and 4-amino-4-deoxy-*N*^10^-methylpteroic acid (DAMPA) to some extent, are potentially associated with various toxicities (Klapkova et al., 2011; Schofield et al., 2015). Therefore, establishment of an accurate biochemical quantification methodology aimed at MTX and its metabolites is vital to clinical safety and efficiency.

It is noteworthy that an increasing number of studies have found that RA is a heterogeneous disease with complex molecular network of pathophysiology, while MTX can only target a limited number of proteins. To gain a better therapeutic effect, combination regimens are recommended and lead to better outcomes than with either agent alone. *Tripterygium wilfordii Hook F* (TwHF) is a traditional Chinese medicinal herb, and its extract, tripterygium glycosides (TG_S_), exhibit immunosuppressive and anti-inflammatory effects (Su et al., 2015a). Tripterygium glycosides tablets (TGT_S_) as a prescription medicine, mainly containing TG_S_, have been widely used in China treatment of RA for several decades (Zhang et al., 2010). Besides, early in 2002, the extract of TwHF showed good efficacy in treating RA on U.S. patients (Tao et al., 2002). The therapeutic effects of TGT_S_ may be attributed to their multiple components classified as three major groups, including diterpenoids, triterpenoids, and sesquiterpene alkaloids (Liang et al., 2015; Su et al., 2015b). In the view of plasma pharmacochemistry, only when the drug is absorbed into the blood can exert its biological activity. Recently, several randomized control trials further indicated that the addition of TGT_S_ may be able to achieve better effectiveness than DMARDs monotherapy (such as MTX) in RA patients (Wang et al., 2017). Actually, MTX plus TGT_S_ has been empirically used on treatment of RA in China for decades, while the interferences of drug blood level and synergistic mechanism attributed to their joint treatment remains unknown. To avoid the probable danger that drug combination can lead to a shift toward the production of another contributing to the undesirable side effects. It is essential to establish a quantitative and comprehensive monitor strategy on the variation of bioactive migratory constituents and terminal metabolic phenotypes.

Recent advances in metabolomics offer us a chance to identify changes in the concentration of small endogenous metabolites in biological samples caused by drug combination therapy and to speculate on the possible mechanism of drug combination treatment. The pseudotargeted metabolomics method integrated the advantages of both untargeted and targeted methods, is demonstrated to be a comprehensive strategy with high-quality and information-abundant data (Shu et al., 2017). The ultra-high-performance liquid chromatography Q Exactive hybrid quadrupole-orbitrap high-resolution accurate MS (UHPLC-QE Orbitrap HRMS) instrument is very important for *in vivo* metabolomics studies regarding to the identification of untargeted metabolites in complex matrices, and is capable of analyzing a broad range of metabolites by database. For targeted metabolites' quantification, multiple reaction monitoring (MRM) performed on a triple quadrupole tandem mass spectrometry (QqQ-MS) is recognized as the gold standard, attributed to its wide linear dynamic range, high sensitivity, and high repeatability. In summary, the pseudotargeted metabolomics gives us a new chance to study the possible mechanisms of drug combination therapy.

Here, we conducted this study, 12-week, a randomized controlled clinical trial, integrating clinical biochemical test indicators, quantitative plasma pharmacochemistry and the pseudotargeted metabolomics, to monitor the variation of drug blood level and perturbation of metabolites caused by MTX plus TGT_S_ combined to treat RA patients. The present clinical research can assist in elucidating the polypharmacological and synergistic mechanisms underlying the therapeutic effect of MTX combined TGT_S_ regimen for active RA.

## Patients and methods

### Study design and clinical management

In this 12-week clinic trial, all RA patients, from Beijing China-Japan Friendship Hospital, were enrolled from February, 2016 to April, 2017. Forty-four patients met the following criteria: (1) 18–70 years of age; (2) active disease [active RA was defined by the following performance: ≥4 swollen joints count (SJC), ≥ 6 tender joints count (TJC), morning stiffness lasted at least 30 min, an erythrocyte sedimentation rate (ESR) concentration of ≥ 28 mm/h and/or an C-reactive protein (CRP) concentration of > 20 mg/L]; (3) non-pregnancy and lactation; (4) no alcohol abuse, no other serious concurrent illnesses or recent major surgery; (5) no received DMARDs or glucocorticoids therapy within 3 months before participating in this study. We divided the patients by 1:1:1 ratio into three treatment groups, specific as follows. 14 patients received oral administration of the 20 mg TGT_S_ thrice-daily, while 15 patients received oral MTX tablets at a dose of 7.5 mg in the first week and a dose of 10 mg in the second week, while a dose of 12.5 mg from the third week to the last week. Besides, 15 patients received oral TGT_S_ plus MTX tablets at the same dosage as above. MTX tablets were provided by Shanghai Xin Yi Pharmaceutical Co., Ltd. (National Yaozhun: H31020644). TGT_S_ purchased from Zhejiang De-eng De Pharmaceutical Co., Ltd. (National Yaozhun: Z33020422). No other drugs for treating RA were allowed.

A total of 11 age-matched healthy volunteers were selected as normal controls. Blood samples were collected at baseline (before starting treatment) and 12 weeks after treatment. This study was registered with Beijing China-Japan Friendship Hospital Clinical Trials Registry (ChiCTR-RNC-14004887), and all protocols involving human subjects were approved by the ethics committee of People's Hospital of Yichun (ethics ID: 2014-01). Written informed consent was obtained from each participant.

### Clinical assessments

Efficacy measures included the proportion of patients achieving an ACR 50 (American College of Rheumatology criteria) response and clinical remission [defined as Disease Activity Score (DAS28) <2.6] at week 12. The criteria of ACR 50 was ≥50% improvement in the number of tender and swollen joints (28 tender and 28 swollen joints were assessed), and ≥50% improvement at least 3 of the following 5: (1) the physician's global health status assessment of the patient of disease; (2) the patient's global health status assessment of disease; (3) Visual Analogue Score (VAS) evaluated by the patients for pain; (4) the patient's assessment of function using Health Assessment Questionnaire (HAQ); (5) acute phase reactions (the ESR or plasma CRP level) (Lv et al., 2015). Per cent improvement for each indicator = (*post-treatment value*–*pre-treatment value*)/*pre-treatment value* × 100%. Compared with the DAS28 based on CRP, the DAS28 based on ESR has been extensively validated for its use in clinical trials. Therefore, in this study, DAS28 based on ESR was used to monitor disease activity in RA. DAS28 = 0.56 × SQRT(TJC28) + 0.28 × SQRT(SJC28) + 0.014 × GH + 0.70 × ln(ESR), where GH = growth hormone. Meanwhile, change in the morning stiffness, anti-cyclic citrullinated peptide antibody (Anti-CCP) positive rate, and rheumatoid factor (RF) positive rate were also noted.

### Laboratory measurements

#### Chemicals and reagents

LC-MS (Optima) grade water, methanol, acetonitrile, and formic acid, were purchased from Fisher Scientific (Fair Lawn, NJ, USA). Ammonium hydroxide (25% v/v**)**, ammonium formate, dithiothreitol, and ascorbic acid were of analytical grade. Standard substances of triptolide, tripterine, and wilforlide A were supplied by Chengdu Must Bio-Technology Co., Ltd. The reference standards of demethylzeylasteral, triptonide, triptophenolide, adenosine, guanine, cytosine, uracil, tryptophan, threonine, carnitine, histidine, taurine, methionine, aspartate, glycine, alanine, hypoxanthine, lactic acid, and uric acid were obtained from Shanghai Source Leaf Biological Technology Co., Ltd. Standard substances of wilforol A, wilfortrine, wilforgine, wilforine, wilfornine A, wilfordine, wilformine were purchased from Beijing FuFan Biological Technology Co., Ltd. MTX, MTXPG2-5, 7-OH MTX, DAMPA, 5-methyltetrahydrofolate (5-Me THF), 5-formyltetrahydrofolate (5-Fo THF), and S-adenosy-L-homocysteine (SAH) were provided by Toronto Research Chemicals Inc. The purity of the above references was higher than 98%. Blank human plasma was purchased from UTAK Laboratories (Valencia, CA, USA).

#### Drug preparation

Twenty tablets of TGT_S_ were accurately weighed to determine the average tablet weight, ground in a mortar, and mixed thoroughly. A portion of the powder (equivalent to 10 mg of extract) was accurately weighted and transferred to a conical flask, mixed with 10 mL of methanol by ultrasonication for 30 min to dissolve and extract the analytes. Then make up for lost weight and the clear supernatant was transferred to a sample vial after filtration through a 0.22-μm filter membrane. Injection-volumes were 2 μL for RRLC-QqQ-MS analysis. The extraction protocols have been reported in pervious article (Liang et al., 2015).

Twenty tablets of MTX were accurately weighed to determine the average tablet weight, ground in a mortar, and mixed thoroughly. Some powder (equivalent to 2.5 mg of MTX) was accurately weighted and placed in 25 ml stoppered erlenmeyer flask, adding 25 ml of 0.1 M ammonium hydroxide by ultrasonication for 30 min to dissolve, Then make up for lost weight and the clear supernatant was transferred to a sample vial after filtration through a 0.22-μm filter membrane. Injection-volumes were 2 μL for RRLC-QqQ-MS analysis. The extraction protocols were based on the experiment of Den et al. (2013).

#### Plasma sample collection and preparation

Blood samples were collected in heparinized tubes from all RA patients at baseline (before starting treatment) and 12 weeks after treatment in the morning after an hour of oral administration. Blood samples from normal volunteers were also collected. The plasma was separated from blood samples by centrifuging at 10,000 rpm for 15 min and was instantly frozen at −80°C for the next analysis. Each 1 mL aliquot of plasma sample was mixed with 3 mL of methanol and vortexed for 60 s to precipitate protein. After centrifugation at 5,500 rpm for 15 min at 4°C. The supernatant was transferred and concentrated to dryness at 35°C. The dried residue was then re-dissolved in 100 μL of methanol/ deionized water (1:1, v/v) by ultrasound. The sample solution was transferred to an auto-sampler vial after filtration through a 0.22-μm filter membrane. Injection-volumes were 2 μL and 1 μL for RRLC-QqQ-MS and UHPLC-QE Orbitrap HRMS analysis, respectively.

#### Preparation of stocks, calibration standards samples, and quality control samples

The stock solutions of wilforgine, wilformine, wilforol A, wilfordine, wilfortrine, wilfornine A, triptolide, tripterine, wilforlide A, wilforine, demethylzeylasteral, triptophenolide, triptonide, and adenosine were prepared in methanol at concentrations of 0.93, 1.03, 0.37, 0.38, 0.51, 0.95, 1.06, 0.98, 0.024, 0.52, 1.00, 0.47, 0.99, and 0.045 mg/mL, respectively. The stock solutions of MTX, MTXPG2-5, 7-OH MTX, and DAMPA were prepared in 0.1 M ammonium hydroxide at concentrations of 0.49, 0.25, 0.25, 0.33, 0.34, 0.21, and 0.20 mg/mL, respectively. The stock solutions of SAH, 5-Me THF, and 5-Fo THF were prepared in 0.1 M ammonium hydroxide containing 100 ng/ml of DTT and 100 ng/ml of ascorbic acid at concentrations of 0.43, 0.20, and 0.97 mg/mL, respectively, to obtain the primary stock solutions. The stock solutions were prepared at concentrations of 1.00, 0.39, 1.02, 1.01, 1.00, 0.51, 0.10, 0.50, 0.51, 0.019, 0.99, 1.04, 1.01, 0.21, and 1.00 mg/mL, respectively, for histidine, uracil, carnitine, tryptophan, threonine, taurine, uric acid, cytosine, lactic acid, guanine, methionine, aspartate, glycine, alanine, and hypoxanthine in water. Calibration samples were obtained by a serial dilution with 50% acetonitrile. Quality control sample was prepared by pooling a same volume from each blood sample and then preparing the pooled quality control samples in the same way as the blood samples. All solutions were stored at −20°C prior to use.

### RRLC–QqQ-MS analysis

A Poroshell 120 SB-C18 column (100 mm × 4.6 mm; 2.7 μm) was used for the chromatographic separation to detect the concentration of the drugs and their metabolites. An injected sample volume was 2 μL and the column temperature was set at 30°C The mobile phase A was 10 mM ammonium formate aqueous solution containing 0.1% (v/v) formic acid, and the mobile phase B was acetonitrile containing 0.1% (v/v) formic acid. The gradient elution program (A/B, v/v) was performed as follows. From 0 to 10 min, the mobile phase B was increased from 20 to 80% and the flow rate was 0.3 mL/min. From 10 to 13 min, the mobile phase B was increased from 80 to 100% and the flow rate was increased from 0.3 to 0.5 mL/min. From 13 to 25 min, the mobile phase B was maintained at 100% and the flow rate was 0.5 mL/min. The total run time was 25 min.

A Poroshell 120 HILIC column (100 mm × 2.1 mm; 2.7 μm) was used for the chromatographic separation to detect the concentration of 19 endogenous metabolites in the plasma samples. The mobile phase A was 10 mM ammonium formate aqueous solution containing 0.1% (v/v) formic acid, and the mobile phase B was acetonitrile containing 0.1% (v/v) formic acid, and the gradient program for these samples as follows. From 0 to 6 min, the mobile phase B was maintained at 80%. From 6 to 14 min, the mobile phase B was decreased from 80 to 50%. From 14 to 16 min, the mobile phase B was increased from 50 to 80%, and the mobile phase B was maintained at 80% from 16 to 20 min. An injected sample volume was 2 μL and the column temperature was set at 35°C and eluted at a flow rate of 0.30 mL/min. The total run time was 20 min.

The RRLC-QqQ-MS system consisted of an RRLC system (Agilent Technologies, Palo Alto, CA, USA) including a G1311A binary pump, a G1311A vacuum degasser and a G1311A autosampler and triple quadrupole mass spectrometer equipped with an electrospray source (Series 6410, Agilent Technologies). The mass spectrometer was operated in the ESI positive ion with MRM mode. The MRM quantitative ions were then selected from the MS/MS data (see Tables S1, S2). The optimized conditions of the ESI source were as follows: capillary voltage, 4,000 V; nebulizer pressure, 45 psi; drying gas temperature, 300°C; drying gas, 11 L/min; corona current, 10 Na; sheath gas temperature, 250°C and sheath gas, 7 L/min.

### UHPLC-QE orbitrap HRMS analysis

The untargeted metabolomics analysis was performed using an UltiMate 3000 Hyperbaric LC system coupled to a Q Exactive MS. Chromatographic separation was performed using an Acquity UPLC CSH C18 column (1.7 μm 100 × 2.1 mm). The mobile phase A was water contained 2 mM ammonium formate, v/v, 0.1% formic acid, and the mobile phase B was methanol. From 0 to 6 min, the Phase A was increased to 100% from 25%. From 6 to 20 min, the Phase A was maintained at 100%. The temperatures of the autosampler and column oven were set at 4 and 40°C, respectively, and the flow rate was 0.4 mL/min. The injection volume was 1 μL.

The ESI source was operated in the positive mode with the following parameters: capillary temperature, 350°C; source voltage and spray voltage, 3.7 kV; sheath gas (nitrogen) flow, 28 arb; and aux gas flow, 8 arb. Data were acquired using full MS scan (resolution: 70,000; AGC target: 1 × 106; maximum IT: 120 ms; scan range: *m/z* 30–1500). Collision induced dissociation-based data dependent on MS/MS (resolution: 175,00; AGC target: 1 × 105; maximum IT: 120 ms; loop count: 5; top N = 5; isolation window: *m/z* 1.0; scan range: *m/z* 30–1,000; NCE/stepped NCE: 30, 40, 50; under fill ratio: 1.0 %; intensity threshold: 1.0 × 105; apex trigger: 2–6 s; dynamic exclusion: 10 s).

### Method validation

#### Selectivity

The selectivity of the method was determined by comparing the chromatograms of a blank human plasma sample, a blank human plasma sample spiked with the working solutions, the extract of TGTs, the extract of MTX tablets, and the human plasma samples after oral administration of drugs.

#### Linearity and lower limit of quantification (LLOQ)

The calibration curves of the tested compounds were determined by least-squares linear regression of the peak area versus the concentrations. Linearity was expressed as the expected concentration against the measured concentration and was considered acceptable if the squared correlation coefficient (*R*^2^) was >0.99 for each calibration curve. The LLOQ was defined as the lowest concentration, having a signal-to-noise ratio of >10:1.

#### Precision and accuracy

The precision was calculated as RSD (%) within ± 15% and the accuracy was evaluated as RE (%) within the nominal value of 85–115%. Intra-day precision and accuracy were evaluated by replicate analyses of six quality control samples on the same day at three different concentrations. The inter-day precision and accuracy was determined on three consecutive days.

#### Recovery and matrix effect

Recovery of the sample preparation was determined by spiking 3 different batches of blank human plasma with each of compounds before and after sample preparation. The recovery was calculated as recovery (%) = sample spiked before preparation/sample spiked after sample preparation × 100%. The matrix effect was determined by comparing the peak response of the pure standard prepared in the flow phase with the peak response of the analyte in the plasma sample.

#### Stability

The stability of the analyte in stored plasma was studied by measuring the stored quality control samples after 1 months at −80°C. Stability after sample preparation was studied by preparing each of the analyte at three concentrations and measuring this directly and after storage in the autosampler at room temperature for 24 h. Freeze/thaw stability were evaluated using quality control samples after three successive cycles of freezing at −80°C. All results from these experiments were compared with results from freshly prepared and measured samples.

### Statistical analysis

The data obtained from the *UHPLC-QE Orbitrap HRMS* were introduced into the SIMCA-P 13.5 software (Umetric, Umeå, Sweden) to perform the multivariate statistical analysis. Principal component analysis (PCA) is an unsupervised pattern recognition method that is used for analyzing, classifying and reducing the dimensionality of numerical datasets in multivariate problems. The data were subjected to PCA to visualize general clustering, trends or outliers among the observations. Orthogonal partial least squares-discriminant analysis (OPLS-DA) was a supervised discriminant analysis and statistical method, which was utilized to validate the PCA model and identify the differential metabolites. R^2^ represented the explanation capacity of the model and R^2^X represent the fraction of the variance of X matrix, whereas Q^2^ suggested the predictive accuracy of the model. The cumulative values of R^2^X, Q^2^ close to 1 meant an excellent model. The differential metabolites were selected by the statistically-significant threshold of the variable influence on projection (VIP) values. Meanwhile, ROC analysis is a useful tool for evaluating the accuracy of a statistical model. Thus, ROC analysis was carried out to reconfirm the differential metabolites. The significance level of the metabolite differences between groups was calculated by Student's *t*-test using the SPSS software (Version 22.0, International Business Machines Corp., Armonk, NY, USA). Results were considered significant when the *p*-value was<0.05.

## Results

### Patient characteristics

In this study, 44 eligible patients were finally enrolled. All patients had active disease, as reflected by the number of TJC and SJC, ESR, and CRP values and morning stiffness. The RA patients were randomized into three treatment groups: 15 patients received MTX monotherapy, 14 patients received TGT_S_ monotherapy, and 15 patients received the combination therapy. As shown in Table 1, patient demographic and clinical details were no statistically significant differences among the three treatment groups (*p* > 0.05).

**Table 1:** Baseline demographic and clinical characteristics of the 44 rheumatoid arthritis patients enrolled in the study.

**Characteristics**	**MTX group**	**TGT_S_ group**	**MTX + TGT_S_ group**
	**(*n* = 15)**	**(*n* = 14)**	**(*n* = 15)**
Age (SD), years	53.7 (14.2)	54.1 (14.0)	55.5 (13.0)
Women, *n* (%)	28 (80%)	29 (82.8%)	28 (80%)
Weight (SD), kg	59.9 (10.1)	60.3 (11.0)	61.1 (10.7)
Smoking history (SD), years	No	No	No
DAS28 (SD)	5.91 (1.34)	6.12 (1.23)	6.07 (1.27)
Duration of RA (SD), months	70.9 (92.8)	63.7 (89.3)	56.6 (93.1)
Morning stiffness (SD), minutes	97.8 (63.8)	104.7 (57.5)	111.4 (60.0)
Anti-CCP positivity, *n* (%)	27 (77.1)	29 (82.8)	28 (80)
RF positivity, *n* (%)	28 (80)	27 (77.1)	30 (85.7)
TJC (SD), *n*	14.7 (6.0)	15.3 (7.0)	15.9 (6.1)
SJC (SD), *n*	10.6 (6.5)	10.6 (6.5)	11.4 (7.9)
CRP (SD), mg/L	33.3 (28.7)	31.7 (33.2)	38.0 (25.5)
ESR (SD), mm/h	54.3 (26.1)	51.7 (24.4)	57.0 (27.2)
Visual Analogue Score (VAS) evaluated by the patients for pain (SD), mm	69.0 (18.4)	70.6 (23.4)	71.9 (22.5)
The patient's global health status assessment of disease (SD), mm	67.7 (23.7)	65.0 (25.3)	68.4 (19.9)
The physician's global health status assessment of the patient of disease (SD), mm	63.2 (22.2)	61.8 (20.2)	64.0 (24.6)
HAQ (SD)	1.50 (0.53)	1.74 (0.77)	1.73 (0.60)

*Data are presented as mean (SD) or n (%). DAS28,28-joint count Disease Activity Score; Anti-CCP, anti-cyclic citrullinated peptide antibody; RF, rheumatoid factor; TJC, tender joint count; SJC, swollen joint count; CRP, C-reactive protein; ESR, erythrocyte sedimentation rate; HAQ, Health Assessment Questionnaire*.

### Clinical efficacy

**Figure 1:**
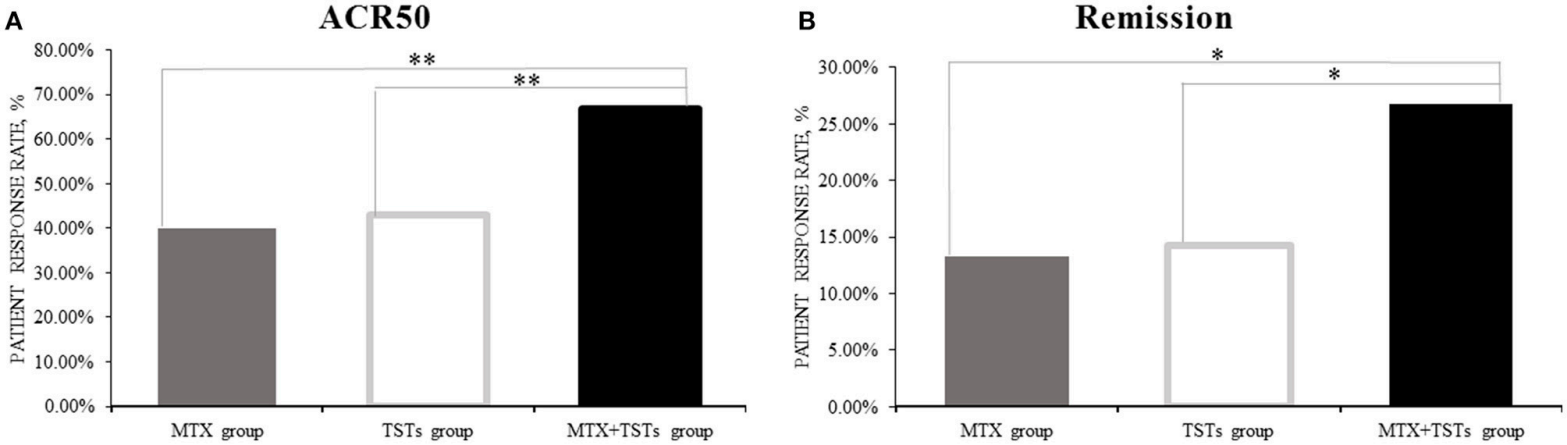
The clinical efficacy of the three groups in week 12. **(A)** ACR50; **(B)** Remission. The percentage of the response was calculated with the denominator of the total number of patients in each group [15,14,15 for methotrexate (MTX) group, tripterygium glycosides tablets (TGT_S_) group, and the combination group, respectively] and the number of patients who reached the response criteria was calculated as the numerator. ACR, American College of Rheumatology. Remission, defined as Disease Activity Score (DAS28) < 2.6. *p < 0.05, **p < 0.01.

Comparison of the data obtained from clinic, at week 12, 40.0% (6/15), 42.9% (6/14), and 66.7% (10/15) of patients who received MTX, TGT_S_, and combination therapy, respectively, reached at least 50% improvement of disease activity as determined by the ACR 50 response criteria. Comparing TGT_S_ monotherapy group to MTX monotherapy group, there was no statistically significant difference (MTX vs. TGT_S_, *p* > 0.05). However, there were significant differences between the combination therapy group and the monotherapy group in terms of ACR 50 response (Figure 1A). Patients who received MTX monotherapy, TGT_S_ monotherapy, and combination therapy reached clinical remission rate of 13.3, 14.3, and 26.7%, respectively. The significant difference was seen in Figure 1B. There was no statistically significant difference in the mean change of HAQ from baseline to week 12 among the three groups (mean improvement was 0.66, 0.76, and 0.83, respectively; *p* > 0.05), as shown in Table 2.

**Table 2:** Clinical efficacy measures of the three groups.

**Paramater**	**MTX group**		**TGT**_**S**_ **group**		**MTX** + **TGT**_**S**_ **group**	
	**0 week**	**12 week**	**0 week**	**12 week**	**0 week**	**12 week**
DAS28 (SD)	5.91 (1.34)	4.35 (1.16)	6.12 (1.23)	4.24 (1.51)	6.07 (1.27)	3.98 (1.28)
TJC (SD), *n*	14.7 (6.0)	7.5 (6.3)	15.3 (7.0)	7.9 (6.7)	15.9 (6.1)	6.6 (5.6)
SJC (SD), *n*	10.6 (6.5)	4.2 (5.3)	10.6 (6.5)	4.3 (5.1)	11.4 (7.9)	3.2 (4.0)
CRP (SD), mg/L	33.3 (28.7)	14.6 (24.2)	31.7 (33.2)	12.1 (20.0)	38.0 (25.5)	12.3 (27.1)
ESR (SD), mm/h	54.3 (26.1)	34.1 (26.3)	51.7 (24.4)	32.6 (29.3)	57.0 (27.2)	30.2 (19.9)
Visual Analogue Score (VAS) evaluated by the patients for pain (SD), mm	69.0 (18.4)	44.6 (21.0)	70.6 (23.4)	42.2 (19.1)	71.9 (22.5)	36.8 (24.2)
The patient's global health status assessment of disease (SD), mm	67.7 (23.7)	41.4 (27.5)	65.0 (25.3)	42.3 (17.8)	68.4 (19.9)	34.9 (22.7)
The physician's global health status assessment of the patient of disease (SD), mm	63.2 (22.2)	38.1 (20.6)	61.8 (20.2)	35.9 (25.1)	64.0 (24.6)	29.7 (28.3)
HAQ (SD)	1.50 (0.53)	0.84 (0.92)	1.74 (0.77)	0.98 (0.99)	1.73 (0.60)	0.90 (0.78)

*Data are presented as mean (SD). DAS28,28-joint count Disease Activity Score; TJC, tender joint count; SJC, swollen joint count; CRP, C-reactive protein; ESR, erythrocyte sedimentation rate; HAQ, Health Assessment Questionnaire*.

### Quantification of ingredients migrating to blood using RRLC-QqQ-MS

Based on the theory of plasma pharmacochemistry, the monitoring of blood drug concentration is extraordinary important for clinical safety and efficiency. In this study, employing RRLC-QqQ-MS system operated in the MRM mode, 20 constituents were measured and quantified, including seven MTX and its metabolites, and 13 TGT_S_ active components.

#### Optimization of RRLC-QqQ-MS conditions

Chromatographic parameters such as column type, mobile phase composition, gradient elution procedure, flow rate of the mobile phase, and column temperature were optimized to obtain the proper separation condition. The Poroshell 120 SB-C18 column achieved the best separation of the 20 reference constituents. The 0.1% (v/v) formic acid solution was proved to give a better response than other concentration (e.g., 0.2% formic acid). It was found that 10 mM ammonium formate aqueous solution containing 0.1% formic acid and acetonitrile containing 0.1% formic acid were the most suitable eluting solvent system. Column temperature of 30°C was proved to realize the best separation. The established method enables us to chromatographically separate MTX from its series metabolites as well as separate TGTs mixture, simultaneously.

A Poroshell 120 HILIC column (100 mm × 2.1 mm; 2.7 μm) was used for the chromatographic separation to detect the concentration of endogenous metabolites in the plasma samples. The mobile phase A was 10 mM ammonium formate aqueous solution containing 0.1% formic acid, and the mobile phase B was acetonitrile containing 0.1% formic acid. The flow rate of 0.3 mL/min and column temperature of 35°C exhibited the best retention rates of stationary phase. The established chromatographic conditions were listed in RRLC–QqQ-MS analysis section.

MRM patterns were used to quantify the analytes in patient plasma, MTX tablets, and TGT_S_. Because MRM patterns were superior to other models in targeted analysis. To obtain the protonated molecules and achieve the maximum response of the compound fragment ion peaks, the capillary temperature, the evaporator temperature, the flow rate of the analytes, and the fragmentation energy were optimized. The cationic mode provides better sensitivity for the target compounds.

#### Optimization of UHPLC-QE orbitrap HRMS conditions

Preparation of plasma samples for untargeted metabolomics analysis by UHPLC-QE Orbitrap HRMS. Different mobile phase compositions were screened to obtain LC chromatograms with better peak shape and separation. The water contained 2 mmol/L ammonium formate and 0.1% formic acid (v/v) in aqueous solution was ultimately selected as the mobile phase to obtain sufficiently good performance for these metabolites with good peak symmetry. To acquire better sensitivity for the base ions of most compounds in the QE Orbitrap HRMS spectra, the ionization parameters including capillary temperature, source voltage, spray voltage, sheath gas flow, and aux gas flow were optimized. The optimum conditions for QE Orbitrap HRMS were decided as follows: capillary temperature, 350°C; source voltage and spray voltage, 3.7 kV; sheath gas (nitrogen) flow, 28 arb; and aux gas flow, 8 arb.

#### Method validation

To provide assurance that the system was suitable for use, six pooled quality control samples were run prior to analysis. These pooled quality control samples were also interspersed between every five samples during the analytical run.

#### Linearity and LLOQ

Linearity of the two methods, including the Poroshell 120 SB-C18 column and the Poroshell 120 HILIC column, were respectively evaluated. The lowest concentrations with RSD < 20% were taken as LLOQ and were found to be 12.51 ng/mL for tryptophan, 24.84 ng/mL for threonine, 12.57 ng/mL for histidine, 20.72 ng/mL for taurine, 101.13 ng/mL for methionine, 81.21 ng/mL for aspartate, 70.45 ng/mL for glycine, 58.53 ng/mL for alanine, 0.58 ng/mL for hypoxanthine, 0.91 ng/mL for cytosine, 6.74 ng/mL for guanine, 4.72 ng/mL for adenosine, 800.12 ng/mL for uric acid, 10.46 ng/mL for uracil, 12.71 ng/mL for carnitine, 25.50 ng/mL for lactic acid, 2.29 ng/mL for SAH, 5.89 ng/mL for 5-Me THF, 0.44 ng/mL for 5-Fo THF, 0.31 ng/mL for MTX, 3.94 ng/mL for MTXPG2, 2.67 ng/mL for MTXPG3, 1.83 ng/mL for MTXPG4, 1.56 ng/mL for MTXPG5, 1.01 ng/mL for 7-OH MTX, 0.32 ng/mL for DAMPA, 0.77 ng/mL for wilforol A, 1.12 ng/mL for wilfortrine, 0.80 ng/mL for wilforgine, 0.71 ng/mL for wilforine, 0.33 ng/mL for wilfornine A, 0.36 ng/mL for wilfordine, 0.23 ng/mL for wilformine, 1.50 ng/mL for triptolide, 1.11 ng/mL for tripterine, 2.38 ng/mL for wilforlide A, 0.21 ng/mL for triptophenolide, 0.42 ng/mL for demethylzeylasteral, and 0.78 ng/mL for triptonide, respectively, which can be seen in Tables S3, S4.

#### Selectivity, precision, and accuracy

No endogenous or extraneous peaks interfering with the analytes at the retention time were observed, indicating the specificity of the two methods were acceptable. The accuracy data of the Poroshell 120 SB-C18 column method ranged from 86.3 to 113.9% (RE), and the intra- and inter-day precision were 4.8–14.3% and 3.3–14.6% (RSD). The accuracy data of the Poroshell 120 HILIC column method ranged from 85.9 to 112.7% (RE), and the intra- and inter-day precision were 5.1–13.6% and 3.7–14.4% (RSD). These indicated that the precision and accuracy of these two methods were acceptable.

#### Recovery, matrix effect, and stability

As detailed in Tables 3, 4, the mean recoveries of the analytes of the Poroshell 120 SB-C18 column method were between 85.9 and 111.1% (RSD < 15%), which of the Poroshell 120 HILIC column method were between 87.2 and 113.6% (RSD < 15%). The matrix effect of the Poroshell 120 SB-C18 column method ranged from 86.8 to 112.2% (RSD < 15%), while the Poroshell 120 HILIC column method ranged from 85.9 to 113.5% (RSD < 15%). Thus, it was shown that methanol was a viable and suitable medium for the extraction of analytes and that there was no measurable matrix effect on the ionization of the analytes. Besides, these analytes were all stable in human plasma with accuracy of two methods, respectively, in the range from 95.2 to 103.0% and 96.0 to 104.3% after 24 h in the room temperature (20°C), storage at −80°C for 1 month and three freeze-thaw cycles.

**Table 3:** The accuracy (intra- and inter- day), precision (intra- and inter- day), recovery, matrix effects and stability of Poroshell 120 SB-C18 column for 20 compounds.

**Compounds**	**Concentration (ng/mL)**	**Accuracy (%)**		**Precision (RSD, %)**		**Recovery**		**Matrix effects**		**Auto-sampler**		**At** −**80** ^**°**^**C for**		**Freeze-thaw cycles**	
		**(*****n*** = **6)**		**(*****n*** = **6)**						**for 24 h**		**1 month**			
		**Intra-day**	**Inter-day**	**Intra-Day**	**Inter-day**	**Accuracy (%)**	**Precision (%)**	**Accuracy (%)**	**Precision (%)**	**Mean (%)**	**RSD (%)**	**Mean (%)**	**RSD (%)**	**Mean (%)**	**RSD (%)**
Methotrexate pentaglutamate	6.28	106.0	98.7	11.2	11.4	92.6	10.5	106.8	5.1	97.8	5.4	96.5	9.0	99.0	8.8
	25.13	102.8	97.4	10.6	5.4	91.4	8.5	99.8	9.4	98.6	9.3	99.4	6.5	101.0	7.6
	50.25	99.9	89.8	6.5	13.8	91.2	10.8	101.2	5.1	98.3	7.4	95.7	10.7	96.3	7.8
Methotrexate tetraglutamate	6.19	106.9	92.4	13.3	3.6	90.0	5.7	103.5	6.4	99.7	7.9	97.5	5.2	96.4	5.1
	24.75	109.3	104.1	14.3	11.6	86.6	7.2	103.2	10.4	101.4	9.0	100.8	9.9	98.6	7.6
	49.50	100.0	90.1	9.9	3.9	108.4	8.9	95.9	6.1	98.0	5.0	102.3	8.8	95.4	5.1
Methotrexate triglutamate	6.13	99.3	96.9	6.4	7.8	97.9	8.8	102.3	7.5	99.9	4.1	95.7	4.9	95.9	5.6
	24.50	95.1	96.0	7.1	4.8	103.0	9.0	93.5	5.6	99.6	7.3	97.7	9.1	99.9	10.2
	49.00	108.3	86.3	9.0	7.8	95.5	7.1	96.5	8.1	97.6	6.1	99.2	4.1	96.3	6.9
Methotrexate diglutamate	6.25	96.4	105.1	13.5	12.3	88.0	6.0	107.9	5.9	98.6	10.1	101.3	4.7	97.6	7.5
	25.00	88.5	94.2	8.7	6.9	94.7	5.1	100.5	3.9	100.5	7.6	98.7	10.0	98.3	5.1
	50.00	105.2	96.1	5.5	6.7	90.2	6.0	98.8	9.0	96.4	5.5	101.6	4.0	99.3	10.7
Methotrexate	6.13	97.2	111.8	8.3	12.0	90.3	3.9	92.5	5.0	98.7	6.4	96.5	6.3	97.7	11.0
	49.00	109.3	89.9	6.1	9.2	96.5	11.0	89.3	6.8	101.5	6.6	97.6	4.9	100.1	7.1
	98.00	108.7	112.4	12.6	9.2	111.1	7.6	98.5	9.1	100.6	10.8	102.2	8.5	98.1	4.3
7-Hydroxy methotrexate	3.28	96.4	100.5	5.7	9.0	106.6	5.3	104.6	7.3	102.5	4.6	101.8	9.1	96.5	6.1
	26.25	98.5	92.6	8.9	5.4	97.7	6.3	89.6	7.5	100.3	7.7	102.4	7.4	102.5	10.9
	105.00	90.5	105.9	4.8	3.3	107.1	10.4	97.1	9.6	103.0	9.1	96.2	6.4	102.8	8.0
4-Amino-4-deoxy-N10-methylpteroic Acid	1.51	93.2	107.7	5.9	13.8	105.8	8.7	107.5	5.4	98.3	9.1	97.0	5.6	99.9	4.4
	6.03	92.3	109.1	7.3	5.1	105.8	5.6	92.3	10.2	96.1	9.4	97.6	10.2	97.7	4.8
	12.06	113.9	110.9	8.0	4.0	96.7	8.2	96.0	4.8	101.2	6.2	102.0	10.9	97.1	5.9
Triptolide	26.50	106.2	88.4	13.4	12.4	94.1	7.8	103.0	9.5	102.2	5.2	102.7	4.1	100.4	10.4
	106.00	104.0	96.6	5.8	8.5	107.4	11.2	104.7	8.8	97.5	6.8	99.4	7.0	96.7	5.9
	212.00	96.7	95.9	12.8	12.2	93.1	6.5	99.2	5.3	101.0	9.6	101.6	6.4	99.9	11.0
Triptonide	6.16	101.4	91.2	5.3	12.7	105.4	4.5	105.6	4.4	96.9	5.0	95.6	10.1	95.6	9.1
	24.63	87.7	91.5	10.6	6.9	97.2	6.2	110.8	9.6	99.8	5.3	99.7	4.0	99.2	8.5
	49.25	112.9	89.2	8.8	12.9	88.2	8.5	97.0	9.5	98.8	7.5	95.9	5.2	96.2	4.7
Wilfortrine	25.25	113.9	104.3	9.4	9.1	95.2	9.4	92.8	7.9	102.2	9.3	100.4	7.5	98.1	11.1
	101.00	103.9	96.5	11.2	12.6	85.9	8.7	94.0	7.7	101.5	9.4	101.6	5.6	98.5	4.1
	202.00	99.7	90.5	13.5	12.4	109.4	6.9	110.5	10.0	101.9	3.8	98.5	4.8	102.7	10.5
Wilfordine	1.19	87.2	104.3	12.7	12.2	108.8	10.8	98.7	4.5	96.3	6.2	96.9	9.6	101.6	9.8
	7.13	110.3	106.0	12.4	14.0	107.3	5.8	98.0	7.1	96.4	4.6	100.7	6.2	96.2	9.4
	29.25	98.2	96.3	11.5	5.2	100.0	5.3	91.5	7.8	100.9	4.4	102.1	7.4	97.7	9.2
Wilformine	0.53	91.7	86.9	13.0	7.2	99.7	5.7	110.3	4.3	102.0	10.2	99.9	4.2	101.3	6.7
	1.07	92.4	104.9	11.9	11.0	101.2	11.1	93.0	10.5	101.3	5.4	95.6	9.1	102.0	10.1
	3.22	93.3	97.5	8.2	6.7	96.1	7.3	109.8	7.9	102.6	7.9	98.5	9.1	100.3	5.7
Wilforgine	1.95	101.3	92.9	5.3	6.0	109.3	7.1	104.4	9.2	99.8	7.6	99.1	6.2	97.3	10.1
	23.38	90.8	100.6	5.1	3.8	105.3	6.0	97.9	11.0	96.1	10.7	99.4	5.2	103.0	6.8
	46.75	113.9	101.8	14.1	6.4	110.9	9.8	96.2	8.7	98.1	10.1	100.8	4.5	98.2	5.0
Wilfornine A	0.49	111.1	89.0	11.0	6.9	107.8	6.0	101.3	8.3	99.5	6.0	102.3	10.6	95.7	8.5
	0.98	103.0	107.6	13.8	10.8	107.9	6.9	89.5	7.8	102.2	11.0	97.2	6.5	102.7	4.9
	2.95	104.8	87.8	12.4	6.5	87.2	11.1	100.7	10.3	101.7	10.7	100.2	10.8	98.7	5.3
Triptophenolide	0.50	89.8	96.2	13.4	13.7	88.8	8.0	98.7	10.7	101.8	7.0	96.9	4.2	95.6	10.8
	1.98	105.5	104.1	13.0	6.0	108.3	3.9	103.5	4.9	96.8	4.1	101.2	6.7	101.8	4.1
	5.93	95.0	109.6	9.1	3.5	90.6	8.9	88.1	6.8	101.9	8.2	98.9	8.9	98.5	5.8
Wilforine	1.08	113.6	89.5	10.8	13.1	100.7	5.2	88.2	7.5	98.8	10.7	99.1	10.1	96.0	6.8
	6.45	106.9	99.0	13.7	5.5	104.8	6.1	89.4	8.7	95.9	6.1	98.1	6.2	95.8	9.9
	25.80	90.8	101.8	5.4	10.8	105.5	8.3	112.2	6.9	100.1	6.5	100.9	7.6	98.9	9.7
Demethylzeylasteral	1.04	105.5	110.4	12.6	14.6	93.7	9.4	106.9	7.9	98.9	5.7	102.9	9.4	95.5	10.3
	4.15	89.3	101.6	9.4	5.5	92.2	6.5	92.0	6.5	95.9	4.6	99.4	4.2	101.7	4.1
	12.44	101.1	97.5	8.9	6.9	100.6	10.9	102.1	8.2	95.4	9.1	95.9	6.3	98.4	4.8
Tripterine	6.31	103.3	95.8	14.3	14.2	101.5	7.3	106.1	9.8	103.0	8.5	101.1	11.2	99.6	6.4
	25.25	89.2	97.4	5.5	3.5	93.6	7.1	88.3	6.3	99.5	10.4	102.4	6.9	95.4	9.4
	50.50	94.8	110.1	6.4	5.8	103.9	7.7	92.0	7.1	97.4	5.7	98.6	10.0	98.0	6.0
Wilforlide A	5.00	100.4	103.8	7.3	7.5	90.6	11.0	97.1	9.7	102.7	4.6	95.3	6.2	96.8	8.9
	30.00	104.6	95.5	12.2	9.5	101.7	3.9	93.3	7.5	101.4	10.0	96.2	7.3	97.5	8.7
	60.00	104.1	97.5	10.7	4.7	101.7	7.9	86.8	10.7	101.0	4.2	101.7	9.4	95.2	4.0
Wilforol A	6.25	103.0	90.5	12.4	8.6	106.3	9.3	92.2	4.9	98.3	7.0	97.2	6.4	101.2	4.7
	25.00	88.0	99.3	9.0	9.9	89.2	7.2	89.0	5.6	98.7	5.1	97.8	10.5	97.5	6.4
	50.00	102.2	90.8	13.8	10.5	107.9	6.4	101.3	9.0	97.7	8.1	100.7	8.7	99.2	5.5

**Table 4:** The accuracy (intra- and inter- day), precision (intra- and inter- day), recovery, matrix effects and stability of Poroshell 120 HILIC column for 19 compounds.

**Compounds**	**Concentration (ng/mL)**	**Accuracy (%)**		**Precision (RSD, %)**		**Recovery**		**Matrix effects**		**Auto-sampler for**		**At** −**80** ^**°**^**C for**		**Freeze-thaw cycles**	
		**(*****n*** = **6)**		**(*****n*** = **6)**						**24 h**		**1 month**		
		**Inter-day**	**Inter-day**	**Inter-day**	**Inter-day**	**Accuracy (%)**	**Precision (%)**	**Accuracy (%)**	**Precision (%)**	**Mean (%)**	**RSD (%)**	**Mean (%)**	**RSD (%)**	**Mean (%)**	**RSD (%)**
Lactic acid	1,632.00	94.6	103.6	5.1	9.4	107.2	7.2	95.5	7.6	99.6	9.9	99.0	8.0	102.7	5.4
	40,800.00	112.5	100.0	5.7	9.1	96.6	10.3	85.9	5.6	104.1	3.8	100.0	7.0	98.5	9.5
	91,800.00	104.8	99.8	10.8	5.0	96.0	4.7	105.7	7.7	101.4	9.4	102.7	7.2	102.0	6.6
Hypoxanthine	100.00	103.4	104.3	12.4	12.4	105.0	5.8	96.0	5.3	98.8	6.8	100.1	7.8	102.2	9.6
	400.00	93.8	92.6	10.8	4.1	95.9	5.0	101.4	7.7	101.9	8.0	96.8	6.5	100.1	10.1
	800.00	87.0	107.8	7.2	10.5	112.6	5.7	86.6	5.6	102.7	4.1	98.6	8.7	102.0	5.2
Tryptophan	1,608.00	104.3	109.8	7.1	9.5	107.2	9.0	99.8	5.3	101.0	10.2	103.4	4.8	97.1	10.4
	6,432.00	96.8	103.5	9.1	8.7	101.1	5.1	86.9	5.7	96.6	6.5	99.2	4.0	97.6	9.8
	25,728.00	107.5	110.3	7.1	10.4	95.2	4.7	113.5	6.8	102.5	3.6	102.2	5.2	103.4	8.8
5-Formyltetrahydrofolate	1.21	86.4	106.9	9.9	4.4	90.6	7.4	96.4	3.7	96.1	9.5	97.3	3.8	101.2	10.5
	6.06	104.6	96.4	9.5	6.4	112.6	3.5	109.8	6.8	96.3	9.3	104.2	10.0	96.4	7.1
	12.13	89.6	102.7	9.6	7.6	113.6	3.9	109.9	7.4	100.6	10.5	97.4	9.9	102.7	8.1
Methionine	788.00	96.2	105.4	5.9	8.5	105.8	10.4	89.0	9.2	101.5	8.6	97.2	4.4	99.1	7.3
	4,728.00	103.3	103.5	7.4	11.2	93.5	4.6	100.6	8.8	100.1	3.7	103.2	6.5	103.5	4.7
	9,456.00	101.2	112.4	9.0	3.7	87.5	6.9	94.5	4.2	102.2	4.5	100.8	10.3	99.8	3.6
Uracil	25.50	110.0	98.9	11.5	6.7	109.8	9.6	108.7	9.0	101.3	9.6	98.1	4.5	102.8	4.2
	102.00	112.5	104.7	7.0	7.1	91.1	5.4	103.8	6.3	97.3	10.4	103.2	7.0	100.7	6.8
	204.00	102.5	86.6	8.7	5.6	103.5	5.5	92.1	8.0	103.5	6.2	100.6	7.1	96.0	7.3
Guanine	18.00	88.8	93.8	10.9	10.9	103.4	4.6	87.8	6.0	101.5	5.8	99.0	5.8	97.8	9.0
	72.00	97.8	91.5	8.6	4.4	96.7	5.3	101.6	4.9	100.5	10.5	97.4	5.3	101.9	4.6
	144.00	94.3	86.0	11.9	6.2	87.4	3.6	107.7	3.7	103.9	9.9	103.1	3.7	97.3	10.0
Taurine	1,262.50	110.1	98.5	11.0	4.0	101.3	8.8	89.5	10.6	102.5	5.7	97.4	4.9	97.8	6.4
	10,100.00	112.3	109.5	6.8	9.4	95.3	9.1	91.0	5.2	96.8	4.7	98.2	4.4	96.0	6.6
	15,150.00	93.8	106.5	10.8	4.3	112.3	6.4	109.0	6.5	97.7	7.4	96.3	7.8	101.1	9.6
Adenosine	28.25	100.3	105.9	13.6	12.6	99.7	8.5	101.5	4.6	97.0	5.1	97.9	5.5	103.7	10.4
	113.00	99.4	85.9	12.7	8.7	111.4	4.2	99.2	3.9	103.7	5.2	104.1	4.2	100.6	4.8
	226.00	87.4	111.5	10.4	11.9	113.0	10.6	108.2	10.1	103.3	10.4	97.6	6.6	98.4	8.5
Uric acid	6,400.00	99.3	110.1	6.7	10.4	106.3	5.5	90.9	7.7	99.2	6.7	97.5	10.4	96.9	10.4
	51,200.00	94.5	109.7	12.4	6.6	109.7	5.7	110.6	7.6	98.1	9.2	96.8	7.2	97.7	9.9
	102,400.00	95.4	110.4	6.8	8.0	92.4	8.2	109.6	4.2	102.1	9.2	96.9	7.7	100.8	8.7
Threonine	1,592.00	87.0	109.3	10.2	11.8	87.9	6.6	104.0	10.3	101.3	8.5	98.3	5.5	102.8	7.8
	6,368.00	93.4	95.9	8.0	11.4	109.2	4.2	90.0	9.0	101.1	5.1	103.4	5.1	96.7	10.2
	19,104.00	94.8	86.4	6.4	7.2	107.3	7.2	88.9	4.4	99.8	8.0	99.0	3.8	99.7	7.7
Aspartate	325.00	110.3	98.9	13.0	12.4	96.7	5.9	87.9	6.1	103.3	8.7	99.9	9.4	100.3	4.4
	1,300.00	91.3	94.2	10.7	6.2	90.6	7.8	95.1	8.6	100.2	5.7	99.5	9.3	102.2	3.7
	2,600.00	98.5	98.9	6.9	4.4	94.5	10.5	99.3	4.9	97.5	10.5	98.1	3.8	103.1	10.0
Alanine	5,670.00	104.0	103.2	9.2	6.6	107.7	10.1	101.5	9.8	96.1	8.9	99.7	10.2	98.5	10.5
	22,680.00	100.4	100.4	7.4	5.4	109.9	7.3	100.7	5.0	98.2	4.1	104.3	10.3	96.9	10.1
	45,360.00	87.0	108.3	8.4	7.6	102.7	6.6	89.8	10.1	97.7	5.5	100.2	3.5	102.6	5.3
Glycine	1,616.00	97.4	101.2	9.8	8.8	101.8	6.8	104.6	6.5	97.0	8.0	102.6	5.3	97.4	9.8
	12,928.00	107.4	89.0	7.4	9.7	110.3	6.5	90.4	9.4	102.0	10.7	103.0	5.7	98.4	7.0
	19,392.00	97.6	94.9	11.9	9.2	102.5	6.7	92.8	7.6	102.0	3.9	97.2	6.7	97.4	8.7
Carnitine	1,015.00	103.0	93.2	6.5	8.4	107.9	10.4	91.7	8.1	100.4	7.8	102.8	8.7	99.6	6.4
	4,060.00	104.0	86.2	11.1	14.4	101.6	7.4	108.4	7.6	97.0	7.2	102.2	9.2	100.1	7.1
	9,135.00	106.4	112.0	8.8	5.1	110.8	8.5	102.5	7.0	100.4	5.4	97.5	5.7	98.4	3.5
Cytosine	24.75	104.3	109.7	11.0	13.1	108.5	4.8	93.9	4.2	97.3	7.6	98.0	7.3	98.7	5.1
	99.00	91.2	94.1	12.2	13.0	98.0	8.2	102.6	5.9	103.0	8.1	102.3	6.5	103.9	8.6
	198.00	98.3	111.8	5.3	9.2	95.6	8.7	99.4	4.1	102.6	8.1	96.8	9.3	96.5	10.7
5-Methyltetrahydrofolate	25.00	98.0	92.4	11.3	7.0	88.2	8.2	94.3	6.8	103.6	8.5	98.1	10.2	102.5	5.5
	100.00	102.8	93.4	5.2	10.2	107.6	4.1	104.5	6.3	100.6	4.2	102.8	6.0	98.1	9.0
	200.00	107.1	111.7	12.3	9.2	101.6	8.6	91.6	7.0	102.1	6.2	96.6	3.8	102.6	7.1
S-adenosy-L-homocysteine	6.25	94.8	109.5	9.9	11.2	99.0	4.7	95.5	5.5	96.3	6.4	100.3	10.5	101.5	7.2
	25.00	91.4	96.3	6.8	9.9	100.9	8.7	94.5	10.7	96.8	6.8	98.5	4.3	96.8	10.2
	50.00	112.7	100.9	6.0	5.6	103.0	4.5	93.1	3.5	102.0	10.6	102.2	8.4	101.0	9.6
Histidine	400.00	100.3	88.8	12.4	7.4	87.2	8.7	97.2	8.5	99.6	8.4	98.6	7.4	99.6	7.2
	6,400.00	90.8	97.0	8.8	9.6	94.5	8.7	91.0	3.6	103.3	4.4	100.8	4.1	97.0	8.1
	25,600.00	110.1	96.4	9.3	12.2	94.3	4.5	96.5	9.6	98.4	8.2	102.8	7.2	102.0	10.2

#### Quantification of ingredients of TGT_S_ and MTX tablets

TGTs, a commercial traditional Chinese medicine (TCM), with very potent anti-inflammatory effects and has been used in China for the treatment of RA. The therapeutic effects of TGT_S_ may be attributed to its multiple components, including triptolide, triptonide, triptophenolide, tripterine, wilforlide A, wilforol A, demethylzeylasteral, wilforine, wilforgine, wilfordine, wilformine, wilfornine A, and wilfortrine. Previous studies have also shown that the above-mentioned constituents are the main representative ingredients in TGTs [9, 10]. In this study, the targeted thirteen prototypical compounds, including three diterpenoids, four triterpenoids and six sesquiterpene alkaloids were determined in TGT_S_ from five different batches (Table S7, *p* > 0.05). More details of TGT_S_, including specific batch number, excipients information and average tablet weight were provided in Table S5.

MTX, a folic acid analog, is a first-line treatment and is the most commonly prescribed DMARD for the treatment of RA. In this study, MTX, main contained in MTX tablets, were determined in five different batches, which content ranged from 2.41 to 2.53 mg per tablet from different samples (*p* > 0.05). More details of MTX tablets were provided in Table S6.

#### Quantification of active ingredients in plasma samples of RA patients after mono- or combined oral administration

**Figure 2:**
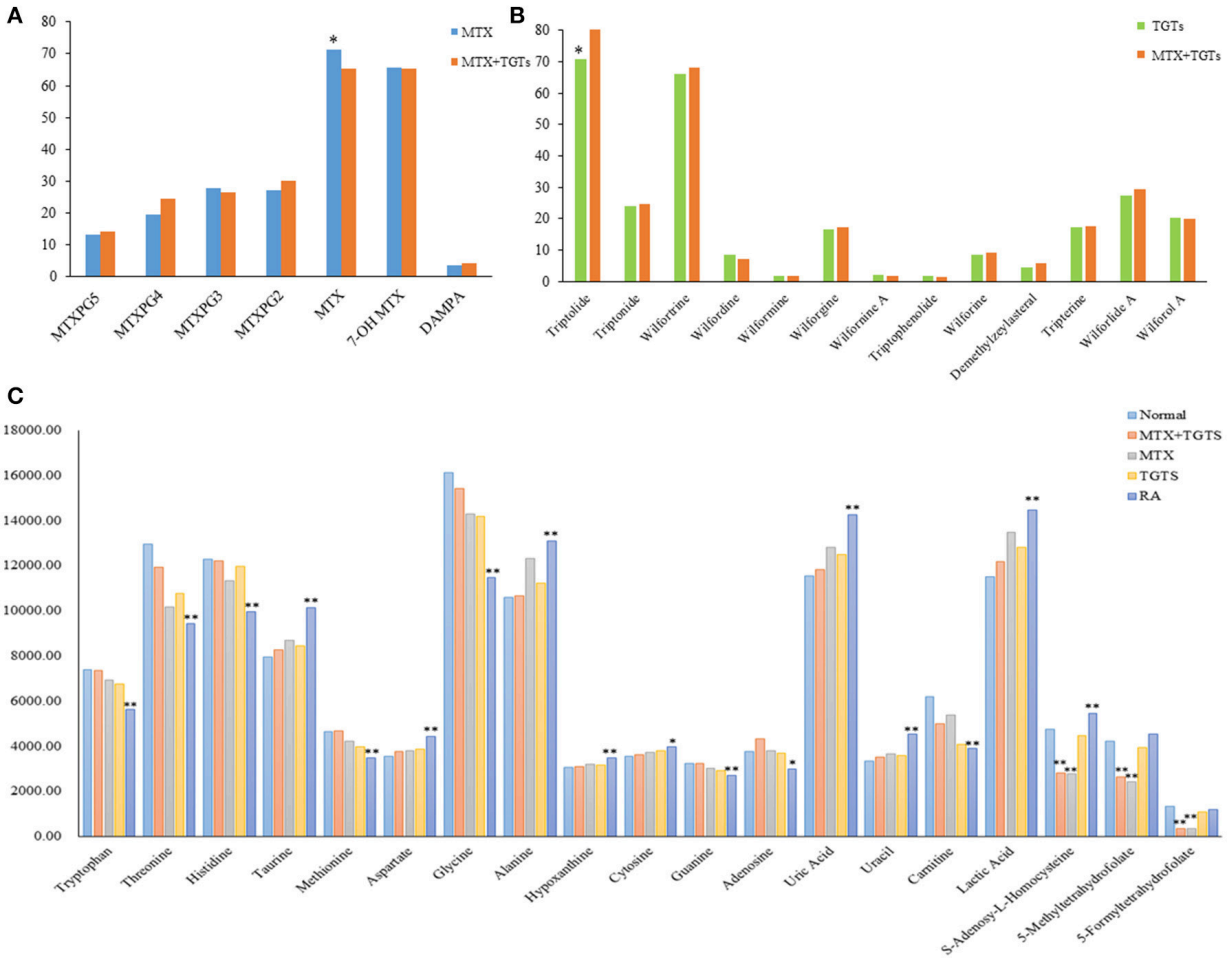
The relative concentrations of 39 compounds. **(A)** The relative concentrations of seven MTX-related compounds in MTX group and MTX plus TGT_S_ group. *p < 0.05. **(B)** The relative concentrations of 13 TGT_S_-related compounds in TGT_S_ group and MTX plus TGT_S_ group. *p < 0.05. **(C)** The relative concentrations of 19 endogenous metabolites in each group. *p < 0.05, **p < 0.01, compared with the normal group.

**Figure 3:**
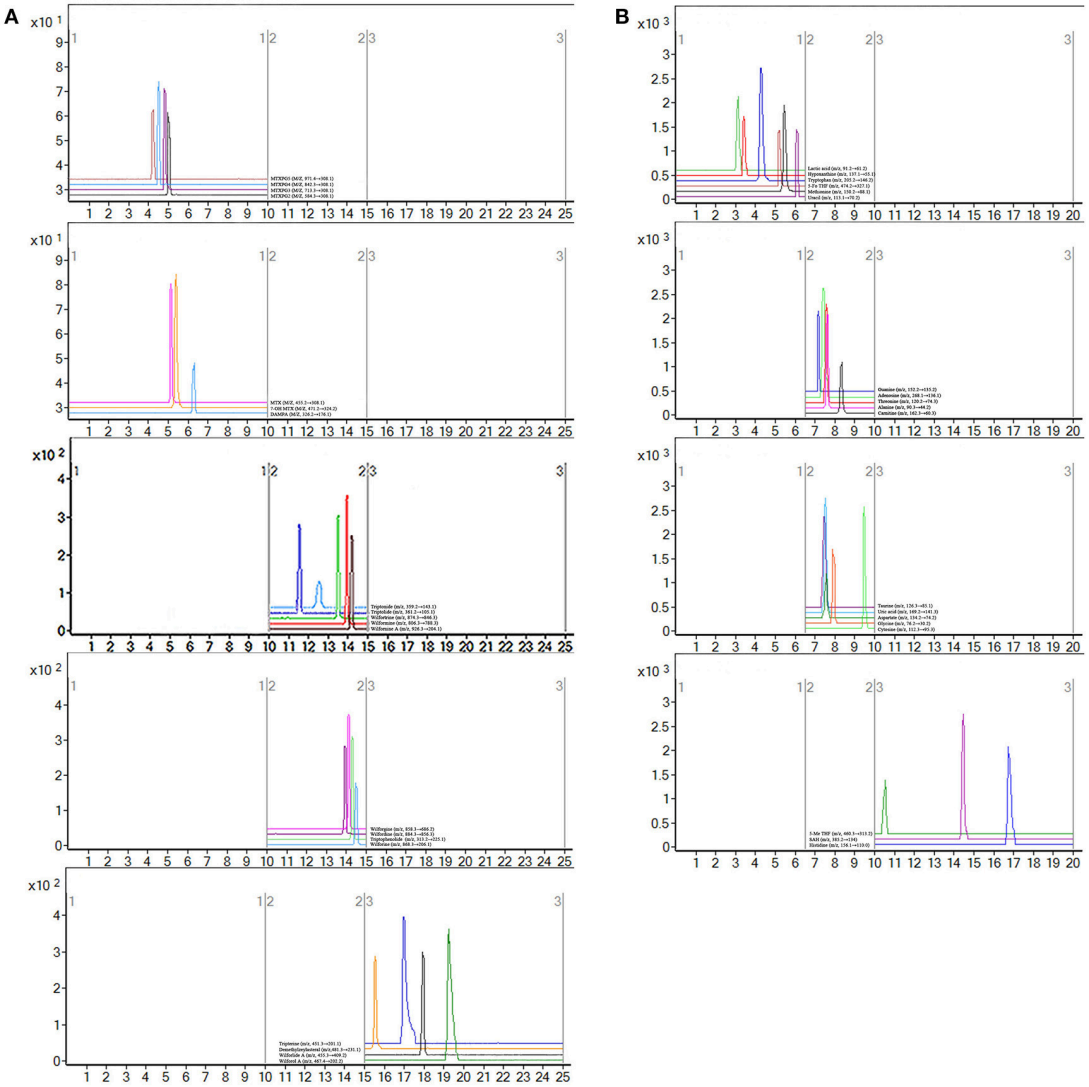
Representative MRM chromatograms. **(A)** MRM figure for 20 compounds, including seven methotrexate (MTX) -related and 13 tripterygium glycosides tablets (TGT_S_)-related; **(B)** MRM figure for 19 endogenous metabolites.

MTX can enter the cell after oral. Once inside the cell, MTX is intracellularly converted to active MTXPGn. MTX is mainly metabolized extracellularly to 7-OH MTX and DAMPA. In this study, the targeted 20 compounds, including seven MTX-related metabolites and 13 TGT_S_-related migratory ingredients, were determined in 44 plasma samples of RA patients after oral administration (Figures 2A,B). Representative MRM chromatograms were shown in Figure 3A. The precise data were shown in Table S8.

### Pseudotargeted metabolomics for screening endogenous metabolites associated with RA

Metabolomics is a scientific discipline focused on the association between disease and metabolic profile in tissue and biological fluids. It can also reflect the therapeutic effect of drugs on the disease, contributed to find potential therapeutic mechanisms for drugs. The pseudotargeted metabolomics method which integrates the advantages of both targeted and untargeted methods, is demonstrated to be a comprehensive strategy with high-quality and information-abundant data.

#### Untargeted metabolomics for locking differential endogenous metabolites

**Figure 4:**
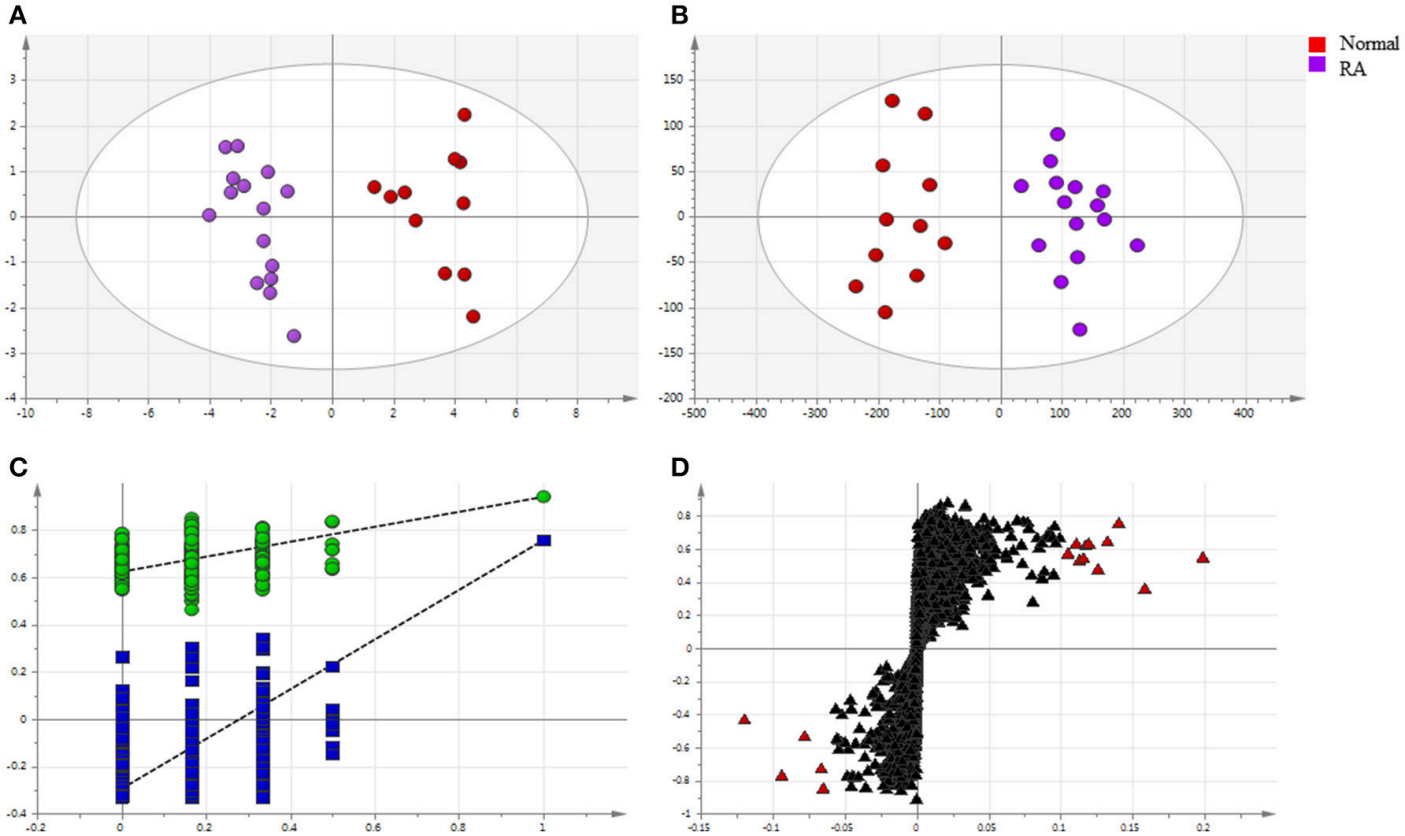
Multivariate statistical analysis of endogenous metabolites in rheumatoid arthritis patients and healthy controls: **(A)** PCA score plot; **(B)** OPLS-DA score plot; **(C)** cross-validation plot of the OPLS-DA model; **(D)** S-plot of OPLS-DA (VIP > 1.0 have been marked red).

Endogenous metabolites in plasma samples of all groups were identified, respectively, using UHPLC-QE Orbitrap HRMS by Progenesis QI software (Waters, Manchester, U.K.), containing multiple MS identification tools for untargeted metabolites identification. Then, the data from UHPLC-QE Orbitrap HRMS were input into the SIMCA-P 13.5 software. PCA and OPLS-DA were utilized to classify the metabolic phenotypes and identify the differentiating metabolites between RA patients and healthy controls. A PCA score plot for first and second principal components was utilized to depict the general variation among the samples of two groups (R^2^X = 0.837, Q^2^ = 0.539), as shown in Figure 4A. Seen from Figure 4B, scores plot of OPLS-DA could divide the RA patients and healthy volunteers into separate blocks. Cross-validation analysis showed that the OPLS-DA model was reliable (Figure 4C). An S-plot was utilized to identify the metabolites according to their contributions to diverse clustering (Figure 4D). Sixteen metabolites were selected for their VIP value greater than 1. As shown in Figure S1, 16 differential endogenous metabolites, with areas under the ROC curves ranging from 0.83 to 1, were considered to show the greatest diagnostic accuracy. Meanwhile, the AUC value of the established model was 1.000, which shown a good ability for discriminating the two groups. The same method were used to identify the differentiating metabolites between RA patients group before and after MTX plus TGT_S_ combination therapy, including the above metabolites, additional three metabolites were selected.

#### Targeted metabolomics for quantifying 19 endogenous metabolites

Notably, evidence implies that RA patients involved in the perturbation of metabolism, including amino acids metabolism, nucleic acid metabolism, lipid metabolism, and oxidative stress. In this study, targeted metabolomics employing RRLC-QqQ-MS system was used to quantify 19 differential endogenous metabolites, including tryptophan, threonine, adenosine, uric acid, taurine, histidine, cytosine, guanine, uracil, carnitine, lactic acid, methionine, aspartate, hypoxanthine, glycine, alanine, 5-Me THF, 5-Fo THF, and SAH. These analytes were measured in each group. Representative MRM chromatograms were shown in Figure 3B. The calibration curves were used for the quantitative determination of the 19 compounds. The precise data were shown in Table S9. The developed and validated RRLC-QqQ-MS method can be effectively applied for simultaneous and quantitative determination of endogenous metabolites in the plasma of patients with active RA.

As shown in Figure 2C, patients with RA had significantly lower levels of a variety of amino acids, including tryptophan, threonine, histidine, methionine, and glycine, than normal people (*p* < 0.01). However, the levels of taurine, aspartate, and alanine were significantly increased (*p* < 0.01). The levels of hypoxanthine, cytosine, uracil, and uric acid increased, conversely, the levels of adenosine and guanine decreased in the plasma of RA patients compared to healthy controls (*p* < 0.05). Meanwhile, the content of carnitine decreased and the content of lactic acid increased (*p* < 0.01). These metabolic products can severely disrupt cellular signaling processes. After administration, the levels of the 16 endogenous metabolites were improved, respectively, in the three groups at week 12. Among them, the MTX plus TGT_S_ group was closest to the normal group. It is noteworthy that there was no significant change in levels of 5-Me THF, 5-Fo THF, and SAH in patients with RA compared to normal people, but their levels were significantly reduced in the MTX group and combined group.

## Discussion

Biological fluids, such as blood, contain a large number of metabolites that may provide valuable information on the metabolism of an organism, and thus concerning its health status (Wang et al., 2012). In the present study, blood samples of all participants were collected and tested at baseline and 12 weeks after treatment. The clinical results of this trial indicate that TGT_S_ plus MTX combination therapy was more effective for the treatment of active RA compared with MTX monotherapy or TGT_S_ monotherapy as shown by ACR50 and DAS28 response criteria. At week 12, the combination therapy resulted in obviously improvement of disease activity, including VAS evaluated by the patients for pain, the patient's and physician's global health status assessment of disease, TJC, SJC, ESR, and CRP. Notably, there was no statistically significant difference between TGT_S_ monotherapy and MTX monotherapy.

The quantitative plasma pharmacochemistry methodology was established upon MTX and its corresponding metabolites, and the constituents in TGT_S_. As reported in some literature that the therapeutic effects of MTX were thought to be mediated by its intracellular levels, including MTXPG2-5, whilst, the toxic effects of MTX were closely related to its extracellular metabolites levels, including 7-OH MTX and DAMPA. So, the clinical determination of MTX and its metabolites is essential to ensure its safety and efficiency. TCM is a complex system that exerts its therapeutic effect through the synergistic activity of multiple characteristic constituents. The multiple components in TGT_S_ may be classified as three major groups including diterpenoids (triptolide, triptonide, triptophenolide), triterpenoids (tripterine, wilforlide A, wilforol A, demethylzeylasteral), and sesquiterpene alkaloids (wilforine, wilforgine, wilfordine, wilformine, wilfornine A and wilfortrine) that are related to clinical response of therapeutic effects of RA. For the constituents absorbed into the blood, which have the opportunity to show bioactivities. In the present study, employing RRLC-QqQ-MS operated in the MRM mode for determination and quantification of 20 constituents, including seven MTX related metabolites and 13 TGT_S_ active components, in the blood of RA patients after oral administration. The results of this study confirmed that the combination of MTX and TGT_S_ did not significantly affect the absorption and metabolism of MTX plus TGT_S_ compared with their monotherapy, respectively. It is known that many critical cellular pathways depend on folate and thus there are many ill-health consequences of folate deficiency (Meadows, 2017). The results showed that the combined administration did not improve the decline of folate caused by MTX. Therefore, it is necessary to use folate as an antidote for MTX to treat RA. All these results implied that MTX plus TGT_S_ for treatment of RA, on the one hand, it did not produce greater toxicity caused by the metabolites of MTX. On the other hand, it also did not interfere with the absorption of the ingredient of TGT_S_.

The analysis of metabolic characteristics may provide a starting point for mechanism investigations into the pathogenesis of RA and the curative mechanism of MTX and TGT_S_ combined to treat RA. In this study, the decreased tryptophan level was observed as one of the most specific metabolic markers for RA compared to normal. Kolodziej (2012) showed that the induction of tryptophan catabolism might help to diminish exacerbated immune responses in the RA. Taurine and hypoxanthine are related to oxidative damage and are the direct scavenger of reactive oxygen radicals. It has been reported that the level of reactive oxygen species in plasma was significantly increased in RA patients (Madsen et al., 2011; Guo et al., 2016). Hence, the increased levels of taurine and hypoxanthine might be the defense mechanism against oxidative damage. Carnitine is essential for lipid metabolism, and it acts as an important transport carrier in the transport of long-chain fatty acids to mitochondria for β oxidation. The reduction of carnitine level indicated that the lipid metabolism of RA patients might be enhanced. The lactic acid content increased, attesting that the anaerobic glycolysis was enhanced during the glucose metabolism in RA patients. Then, pyruvate produced alanine under the action of aminotransferase, which led to an increased alanine. The elevated levels of cytosine, uric acid and uracil, and decreased levels of guanine and adenosine testified the abnormal metabolism of purines and pyrimidines in RA. Meanwhile, the reduction of a variety of amino acids, including threonine, methionine, and glycine demonstrated that protein catabolism was enhanced. This metabolic perturbation was consistent with the hypoxic conditions of the inflamed joint, restricting the diffusion of glucose and large molecules from the blood into the synovium.

**Figure 5:**
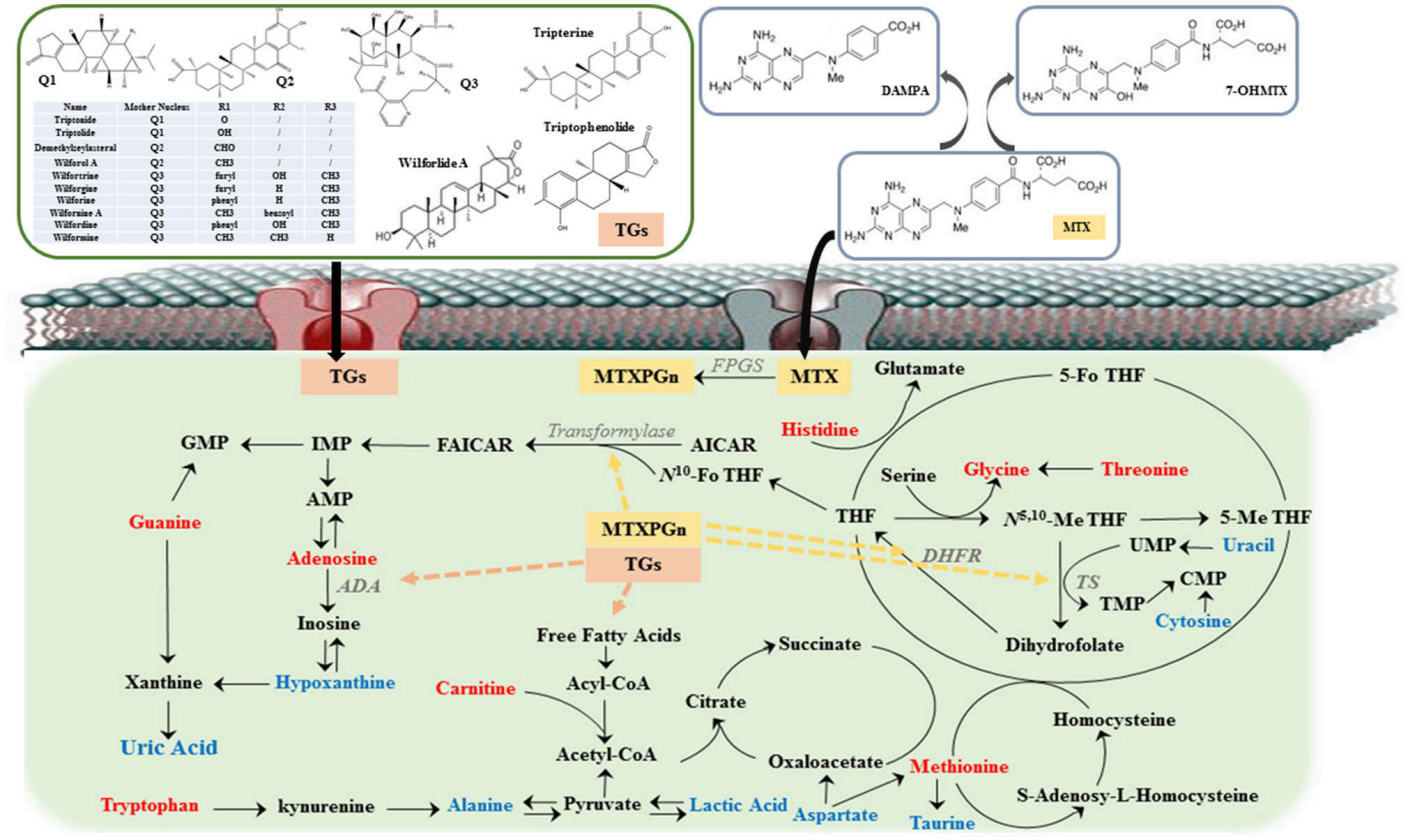
The overview of metabolic imbalances in rheumatoid arthritis (RA) patients and the action mechanism of methotrexate (MTX) combined with tripterygium glycosides tablets (TGT_S_) to treat RA. The color of the font represents the regulation direction of metabolites; the blue represent elevated levels of the RA patients compared to healthy controls; the red represents reduced levels of RA patients compared to healthy controls. Dashed line represents the action site of combination therapy.

According to the results of this trial, the abnormal endogenous metabolites of RA patients were clearly improved in three treatment groups after 12 weeks. Besides, the concentration of 5-Me THF, 5-Fo THF, and SAH had decreased in MTX and combined administration. MTX as a folate antagonist, inhibition of dihydrofolate reductase (DHFR) decreases THF levels, which results in a weakened methionine cycle, inhibition of thymidylate synthase (TS) interference with the conversion of uridylate (UMP) to thymidylate (TMP), and inhibition of 5-aminoimidazole-4-carboxamide ribonucleotide (AICAR) transformylase blocks denovo purine synthesis (Tian and Cronstein, 2007; Taflin et al., 2014; Yuan and Sharer, 2016). All the above, for one thing, may be highly correlated with improvements in nucleic acid metabolism and the reduction of methionine, 5-Me THF, 5-Fo THF, and SAH following MTX therapy. For another, the levels of amino acid improved, including tryptophan, threonine, histidine, taurine, aspartate, glycine, and alanine, might be due to interference with DNA synthesis, which caused T lymphocyte proliferation to be blocked and activated T lymphocyte apoptosis. At the same time, the levels of inflammation and oxidative stress in RA patients were alleviated. Compared with MTX monotherapy group and TGT_S_ monotherapy group, the levels of alanine, adenosine, and lactic acid of the combination group were more closely to the healthy volunteers' group. Wamg Li et al showed that adenosine deaminase (ADA) inhibition by TGT_S_ led to intracellular accumulation of adenosine, which led to the release of adenosine into the extracellular space (Wamg et al., 2007). Adenosine was an important signaling molecule that modulated a large number of physiological functions, the most important of which was anti-inflammation. In addition, the increase of lipid degradation concomitant to ketone body enrichment in the plasma could serve the energy demands of the inflamed joint tissues in RA patients, and the decrease of alanine and lactic acid level suggested that TGT_S_ could effectively improve the energy metabolism disorder of RA (Figure 5).

In the present study, using clinical biochemical test indicators, the theory of plasma pharmacochemistry and the technique of pseudotargeted metabolomics, the potential mechanism of MTX and TGTs combination were preliminarily elucidated. Untargeted metabolomics employing UHPLC-QE Orbitrap HRMS was used to screen the differential endogenous metabolites between RA and healthy controls. Targeted metabolomics employing RRLC-QqQ-MS operated in the MRM mode was used to characterize and quantify 20 drugs metabolites absorbed in the blood and 19 differential endogenous metabolites. The final results showed that TGT_S_ group was as effective as MTX group and the MTX plus TGT_S_ combination group was better than that of their alone groups in RA. To further elucidate the combination mechanisms of MTX plus TGT_S_, deep-going researches on molecular level has been performing in our future studies.

## Author contributions

YL, HF, and CX conceived and designed the experiments. JH provided blood samples and clinical data of rheumatoid arthritis patients. MW analyzed the data and drafted the paper. DH, SZ, and YS took part in the discussion on views in the paper. HL, LL, and SL revised the work critically for important intellectual content.

### Conflict of interest statement

The authors declare that the research was conducted in the absence of any commercial or financial relationships that could be construed as a potential conflict of interest. The reviewer JC declared a shared affiliation, with no collaboration, with the authors, to the handling editor at time of review.

## Abbreviations

RA: rheumatoid arthritis
DMARDs: disease-modifying antirheumatic drugs
MTX: methotrexate
TGT_S_: tripterygium glycosides tablets
MTXPGn: methotrexate polyglutamates
7-OH MTX: 7-hydroxy methotrexate
DAMPA: 4-amino-4-deoxy-*N*^10^-methylpteroic acid
TwHF: *Tripterygium wilfordii Hook F*
TG_S_: tripterygium glycosides
UHPLC-QE Orbitrap HRMS: ultra-high-performance liquid chromatography Q Exactive hybrid quadrupole-orbitrap high-resolution accurate MS
MRM: multiple reaction monitoring
QqQ-MS: triple quadrupole tandem mass spectrometry
SJC: swollen joints count
TJC: tender joints count
ESR: erythrocyte sedimentation rate
CRP: C-reactive protein
ACR: American college of rheumatology
DAS28: disease activity score
VAS: visual analog score
HAQ: health assessment questionnaire
Anti-CCP: anti-cyclic citrullinated peptide antibody
RF: rheumatoid factor
5-Me THF: 5-methyltetrahydrofolate
5-Fo THF: 5-formyltetrahydrofolate
SAH: S-adenosy-L-homocysteine
LLOQ: lower limit of quantification
PCA: principal component analysis
OPLS-DA: orthogonal partial least squares-discriminant analysis
VIP: variable influence on projection
DHFR: dihydrofolate reductase
TS: thymidylate synthase
UMP: uridylate
TMP: thymidylate
AICAR: 5-aminoimidazole-4-carboxamide ribonucleotide
ADA: adenosine deaminase.
