# Supplementary material for: Treatment of Rheumatoid Arthritis Using Combination of Methotrexate and Tripterygium Glycosides Tablets—A Quantitative Plasma Pharmacochemical and Pseudotargeted Metabolomic Approach
Source: Front Pharmacol. 2018 Oct 9;9:1051. doi: 10.3389/fphar.2018.01051 (PMC6189563; doi:10.3389/fphar.2018.01051)
Supplement: Supplementary file 1 [file Table_1.docx]

**Treatment of rheumatoid arthritis using combination of methotrexate and tripterygium glycosides tablets - A quantitative plasma pharmacochemical and pseudotargeted metabolomic approach**

**Captions for Tables**

Table S1. Mass spectral data of 20 compounds.

Table S2. Mass spectral data of 19 compounds.

Table S3. Calibration curves, correlation coefficients, linear ranges and LOQs of 19 compounds

Table S4. Calibration curves, correlation coefficients, linear ranges and LOQs of 20 compounds

Table S5. Details of TGT_S_

Table S6. Details of MTX tablets

Table S7. Contents of 13 constituents in commercial products of TGT_S_ (containing 10 mg extracts)

Table S8. The measurement results of 7 MTX-related and 13 TGT_S_-related ingredients in 44 plasma samples of RA patients after oral administration.

Table S9. The measurement results of 19 endogenous metabolites in each group before and after oral administration.

Figure S1. (A) The ROC curves of the RA/Normal group, ROC curve-based model evaluation (AUC = 1.000). (B) Diagnostic efficacy evaluation using the ROC curves of the biomarkers between two groups. The optimal cutoffs using the closest to top-left corner and the area under ROC curves with a 95% confidence interval are displayed (AUC > 0.83).

Table S1. Mass spectral data of 20 compounds.

| No | Compounds | tR (min) | [M + H]+ (m/z) | MRM Transitions (Precursor ‎→ Product) | Fragmentor (V) | Collision Energy (eV) |
| --- | --- | --- | --- | --- | --- | --- |
| Time segments: 0~10 min | | | | | | |
| 1 | Methotrexate Pentaglutamate | 4.491 | 971.4 | 971.4→308.1 | 160 | 50 |
| 2 | Methotrexate Tetraglutamate | 4.597 | 842.3 | 842.3→308.1 | 180 | 40 |
| 3 | Methotrexate Triglutamate | 4.773 | 713.3 | 713.3→308.1 | 165 | 30 |
| 4 | Methotrexate Diglutamate | 4.936 | 584.3 | 584.3→308.1 | 135 | 30 |
| 5 | Methotrexate | 5.196 | 455.2 | 455.2→308.1 | 148 | 20 |
| 6 | 7-Hydroxy Methotrexate | 5.354 | 471.2 | 471.2→324.2 | 100 | 50 |
| 7 | 4-Amino-4-deoxy-N10-methylpteroic Acid | 6.298 | 326.2 | 326.2→176.1 | 154 | 30 |
| Time segments: 10~15 min | | | | | | |
| 8 | Triptolide | 11.567 | 361.2 | 361.2→105.1 | 158 | 40 |
| 9 | Triptonide | 12.522 | 359.2 | 359.2→143.1 | 135 | 40 |
| 10 | Wilfortrine | 13.505 | 874.3 | 874.3→846.3 | 205 | 30 |
| 11 | Wilfordine | 13.828 | 884.3 | 884.3→856.3 | 162 | 20 |
| 12 | Wilformine | 13.939 | 806.3 | 806.3→788.3 | 160 | 10 |
| 13 | Wilforgine | 14.023 | 858.3 | 858.3→686.2 | 205 | 30 |
| 14 | Wilfornine A | 14.166 | 926.3 | 926.3→204.1 | 120 | 65 |
| 15 | Triptophenolide | 14.28 | 313.2 | 313.2→225.1 | 117 | 20 |
| 16 | Wilforine | 14.431 | 868.3 | 868.3→206.1 | 210 | 45 |
| Time segments: 15~25 min | | | | | | |
| 17 | Demethylzeylasteral | 15.496 | 481.3 | 481.3→231.1 | 134 | 30 |
| 18 | Tripterine | 16.975 | 451.3 | 451.3→201.1 | 100 | 30 |
| 19 | Wilforlide A | 17.901 | 455.3 | 455.3→409.2 | 95 | 5 |
| 20 | Wilforol A | 19.577 | 467.4 | 467.4→202.2 | 100 | 10 |

Table S2. Mass spectral data of 19 compounds.

| No | Compounds | tR (min) | [M + H]+ (m/z) | MRM Transitions (Precursor ‎→ Product) | Fragmentor (V) | Collision Energy (eV) |
| --- | --- | --- | --- | --- | --- | --- |
| Time segments: 0~6.5min | | | | | | |
| 1 | Lactic Acid | 3.001 | 91.2 | 91.2→61.2 | 50 | 12 |
| 2 | Hypoxanthine | 3.376 | 137.1 | 137.1→55.1 | 142 | 34 |
| 3 | Tryptophan | 4.465 | 205.2 | 205.2→146.2 | 86 | 16 |
| 4 | 5-Formyltetrahydrofolate | 5.538 | 474.2 | 474.2→327.1 | 110 | 20 |
| 5 | Methionine | 5.857 | 150.2 | 150.2→88.1 | 95 | 10 |
| 6 | Uracil | 6.018 | 113.1 | 113.1→70.2 | 90 | 14 |
| Time segments: 6.5~10min | | | | | | |
| 7 | Guanine | 7.345 | 152.2 | 152.2→135.2 | 100 | 23 |
| 8 | Taurine | 7.429 | 126.3 | 126.3→85.1 | 120 | 20 |
| 9 | Adenosine | 7.499 | 268.1 | 268.1→136.1 | 96 | 15 |
| 10 | Uric acid | 7.548 | 169.2 | 169.2→141.3 | 110 | 13 |
| 11 | Threonine | 7.634 | 120.2 | 120.2→74.3 | 58 | 6 |
| 12 | Aspartate | 7.661 | 134.2 | 134.2→74.2 | 113 | 14 |
| 13 | Alanine | 7.713 | 90.3 | 90.3→44.2 | 38 | 6 |
| 14 | Glycine | 7.961 | 76.2 | 76.2→30.2 | 85 | 20 |
| 15 | Carnitine | 8.23 | 162.3 | 162.3→60.3 | 116 | 15 |
| 16 | Cytosine | 9.564 | 112.3 | 112.3→95.3 | 103 | 20 |
| Time segments: 10~20min | | | | | | |
| 17 | 5-Methyltetrahydrofolate | 10.423 | 460.3 | 460.3→313.2 | 130 | 20 |
| 18 | S-Adenosy-L-Homocysteine | 14.385 | 385.2 | 385.2→134 | 90 | 20 |
| 19 | Histidine | 16.693 | 156.1 | 156.1→110.0 | 95 | 15 |

Table S3. Calibration curves, correlation coefficients, linear ranges and LOQs of 19 compounds.

| No | Compounds | Calibration curve | r2 | Linear ranges(ng/mL) | LOQ(ng/mL) |
| --- | --- | --- | --- | --- | --- |
| 1 | Tryptophan | y=-4.5773+0.7799x | 0.9947 | 804.00-102912.00 | 12.51 |
| 2 | Threonine | y=-5.4738+1.6080x | 0.9999 | 1592.00-28656.00 | 24.84 |
| 3 | Histidine | y=-0.0102+0.7653x | 0.9985 | 400.00-102400.00 | 12.57 |
| 4 | Taurine | y=1.7777+0.0831x | 0.9988 | 315.63-15150.00 | 20.72 |
| 5 | Methionine | y=-4.6599+2.9934x | 0.9985 | 197.00-9456.00 | 101.13 |
| 6 | Aspartate | y=2.3864+0.1749x | 0.9974 | 162.50-5200.00 | 81.21 |
| 7 | Glycine | y=-2.3747+0.2831x | 0.9988 | 404.00-29088.00 | 70.45 |
| 8 | Alanine | y=-3.8678+1.3602x | 0.9969 | 1890.00-45360.00 | 58.53 |
| 9 | Hypoxanthine | y=9.0754+0.0862x | 0.9979 | 25.00-800.00 | 0.58 |
| 10 | Cytosine | y=0.5265+0.1081x | 0.9999 | 24.75-396.00 | 0.91 |
| 11 | Guanine | y=11.7718+0.9174x | 0.9995 | 18.00-576.00 | 6.74 |
| 12 | Adenosine | y=0.3034+0.2441x | 0.9996 | 14.13-452.00 | 4.72 |
| 13 | Uric Acid | y=-136.1605+27.4916x | 0.9997 | 6400.00-204800.00 | 800.12 |
| 14 | Uracil | y=-6.8511+1.1959x | 0.9967 | 25.50-816.00 | 10.46 |
| 15 | Carnitine | y=6.5355+0.0201x | 0.9986 | 406.00-9135.00 | 12.71 |
| 16 | Lactic Acid | y=-14.8450+2.4934x | 0.9980 | 816.00-91800.00 | 25.50 |
| 17 | S-Adenosy-L-Homocysteine | y=0.8363+1.1323x | 0.9988 | 6.25-200.00 | 2.29 |
| 18 | 5-Methyltetrahydrofolate | y=-36.2065+21.2287x | 0.9975 | 25.00-800.00 | 5.89 |
| 19 | 5-Formyltetrahydrofolate | y=-4.0076+0.5019x | 0.9989 | 1.21-48.50 | 0.44 |

Table S4. Calibration curves, correlation coefficients, linear ranges and LOQs of 20 compounds.

| No | Compounds | Calibration curve | r2 | Linear ranges(ng/mL) | LOQ(ng/mL) |
| --- | --- | --- | --- | --- | --- |
| 1 | MTX | y=-0.2289+0.0473x | 0.9997 | 6.13-392.00 | 0.31 |
| 2 | MTXPG2 | y=3.8184+0.3863x | 0.9985 | 6.25-200 | 3.94 |
| 3 | MTXPG3 | y=2.7451+1.0124x | 0.9958 | 6.13-196.00 | 2.67 |
| 4 | MTXPG4 | y=8.1130+1.1534x | 0.9986 | 6.19-198.00 | 1.83 |
| 5 | MTXPG5 | y=-14.3569+2.6651x | 0.9994 | 6.28-201.00 | 1.56 |
| 6 | 7-OH MTX | y=0.1970+0.4139x | 0.9995 | 3.28-420.00 | 1.01 |
| 7 | DAMPA | y=-3.2130+0.0219x | 0.9962 | 1.51-193.00 | 0.32 |
| 8 | Wilforol A | y=6.1559+2.6182x | 0.9992 | 6.25-200.00 | 0.77 |
| 9 | Wilfortrine | y=-0.0107+0.2003x | 0.9999 | 25.25-404.00 | 1.12 |
| 10 | Wilforgine | y=0.3580+0.1198x | 0.9999 | 1.95-187.00 | 0.80 |
| 11 | Wilforine | y=0.1564+0.0982x | 0.9974 | 1.08-206.40 | 0.71 |
| 12 | Wilfornine A | y=-0.1363+0.0939x | 0.9972 | 0.49-47.25 | 0.33 |
| 13 | Wilfordine | y=-1.4944+0.2312x | 0.9999 | 1.19-234.00 | 0.36 |
| 14 | Wilformine | y=-0.7524+0.0425x | 0.9997 | 0.53-103.00 | 0.23 |
| 15 | Triptolide | y=-9.2715+1.7910x | 0.9982 | 26.50-848.00 | 1.50 |
| 16 | Tripterine | y=7.4213+0.0188x | 0.9936 | 6.31-202.00 | 1.11 |
| 17 | Wilforlide A | y=11.3706+0.0734x | 0.9954 | 5.00-240.00 | 2.38 |
| 18 | Triptophenolide | y=-0.8236+0.1503x | 0.9999 | 0.50-47.40 | 0.21 |
| 19 | Demethylzeylasteral | y=-2.1171+0.1544x | 0.9925 | 1.04-199.00 | 0.42 |
| 20 | Triptonide | y=5.4223+0.9602x | 0.9939 | 6.16-197.00 | 0.78 |

Table S5. Details of TGT_S_

| No | Batch | Average Tablet Weight (mg) |
| --- | --- | --- |
| 1 | 1605119B | 98.90 |
| 2 | 1607103B | 100.14 |
| 3 | 1608112B | 100.09 |
| 4 | 1610101B | 98.53 |
| 5 | 1610102B | 99.71 |

Table S6. Details of MTX tablets

| No | Batch | Average Tablet Weight (mg) | methotrexate (mg/tablet) |
| --- | --- | --- | --- |
| 1 | 036151001 | 85.04 | 2.51 |
| 2 | 036151102 | 84.59 | 2.48 |
| 3 | 036160301 | 84.39 | 2.54 |
| 4 | 036160705 | 84.66 | 2.52 |
| 5 | 036160706 | 84.57 | 2.50 |

Table S7. Contents of 13 constituents in commercial products of TGT_S_ (containing 10 mg extracts).

| Manufacturers | Batch | Content (μg/tablet) | | | | | | | | | | | | |
| --- | --- | --- | --- | --- | --- | --- | --- | --- | --- | --- | --- | --- | --- | --- |
|  |  | Diterpenoids | | | Triterpenoids | | | | Sesquiterpene alkaloids | | | | | |
|  |  | Triptolide | Triptonide | Triptophenolide | Tripterine | Wilforlide A | Wilforol A | Demethylzeylasteral | Wilfortrine | Wilforgine | Wilforine | Wilfornine A | Wilfordine | Wilformine |
| Zhejiang De-eng De | 1 | 6.64 | 3.30 | 4.34 | 58.78 | 37.45 | 0.30 | 25.20 | 142.70 | 37.20 | 28.10 | 4.60 | 11.20 | 15.53 |
|  | 2 | 5.87 | 5.66 | 0.91 | 59.83 | 33.46 | 0.30 | 35.81 | 102.56 | 31.12 | 34.69 | 5.29 | 9.90 | 22.74 |
|  | 3 | 7.99 | 8.71 | 2.82 | 56.49 | 48.94 | 0.40 | 31.57 | 95.70 | 27.67 | 22.73 | 8.75 | 8.55 | 13.92 |
|  | 4 | 5.97 | 4.56 | 1.98 | 58.23 | 39.83 | 0.37 | 27.74 | 133.22 | 30.41 | 33.90 | 4.93 | 8.54 | 21.37 |
|  | 5 | 7.81 | 8.47 | 1.12 | 57.31 | 47.71 | 0.28 | 36.01 | 109.34 | 35.55 | 24.36 | 7.78 | 10.99 | 16.87 |

Table S8. The measurement results of 7 MTX-related and 13 TGT_S_-related ingredients in 44 plasma samples of RA patients after oral administration.

| Ingredients | MTX group (ng/ml) | TGT_S_ group (ng/ml) | MTX+TGT_S_ group (ng/ml) |
| --- | --- | --- | --- |
|  | (n=15) | (n=14) | (n=15) |
| Methotrexate Pentaglutamate | 13.28 ± 13.54 | nd | 14.10 ± 11.49 |
| Methotrexate Tetraglutamate | 19.49 ± 9.21 | nd | 24.57 ± 11.49 |
| Methotrexate Triglutamate | 27.72 ± 13.87 | nd | 26.37 ± 13.85 |
| Methotrexate Diglutamate | 27.25 ± 15.47 | nd | 30.09 ± 14.54 |
| Methotrexate | 71.30 ±33.97 | nd | 65.27 ± 34.06 |
| 7-Hydroxy Methotrexate | 65.54 ± 43.97 | nd | 65.36 ± 41.56 |
| 4-Amino-4-deoxy-N10-methylpteroic Acid | 3.54 ± 2.70 | nd | 4.41 ± 3.05 |
| Triptolide | nd | 110.98 ± 44.18 | 123.74 ± 46.75 |
| Triptonide | nd | 23.87 ± 11.74 | 24.63 ± 11.22 |
| Wilfortrine | nd | 96.25 ± 51.81 | 98.09 ± 45.14 |
| Wilfordine | nd | 8.46 ± 4.71 | 7.12 ± 4.63 |
| Wilformine | nd | 0.70 ± 0.77 | 0.89 ± 0.79 |
| Wilforgine | nd | 16.54 ± 7.83 | 17.28 ± 8.14 |
| Wilfornine A | nd | 1.17 ± 1.12 | 0.86 ± 0.67 |
| Triptophenolide | nd | 0.77 ± 1.01 | 0.44 ± 0.57 |
| Wilforine | nd | 8.46 ± 2.47 | 9.07 ± 3.86 |
| Demethylzeylasteral | nd | 4.57 ± 3.20 | 5.85 ± 3.42 |
| Tripterine | nd | 17.39 ± 4.90 | 17.44 ± 4.81 |
| Wilforlide A | nd | 27.41 ± 8.20 | 29.44 ± 10.33 |
| Wilforol A | nd | 20.23 ± 16.53 | 19.99 ± 15.39 |

Note: nd means not detectable.

Data are presented as mean ± SD.

Table S9. The measurement results of 19 endogenous metabolites in each group before and after oral administration.

| No | Compounds | Before treatment (ng/ml) | | After treatment (ng/ml) | | |
| --- | --- | --- | --- | --- | --- | --- |
|  |  | Normal | RA | MTX | TGT_S_ | MTX+TGT_S_ |
| 1 | Tryptophan | 7380.62±718.03 | 5626.47±586.03 | 6925.90±646.89 | 6756.45±599.72 | 7367.47±606.73 |
| 2 | Threonine | 12945.21±2514.35 | 9427.10±2279.87 | 10167.49**±**2484.36 | 10774.82±2056.89 | 11938.47±2312.91 |
| 3 | Histidine | 12272.43±1110.46 | 9973.01±1858.26 | 11327.47±1492.49 | 11983.68±1342.41 | 12212.18±1673.77 |
| 4 | Taurine | 7963.48±1517.64 | 10134.98±1543.52 | 8691.38±1026.46 | 8437.72±1329.81 | 8267.56±1423.75 |
| 5 | Methionine | 4656.69±726.42 | 3471.97±705.91 | 4234.13±711.15 | 3978.67±643.42 | 4673.13±679.34 |
| 6 | Aspartate | 1768.84±231.64 | 2219.50±301.64 | 1899.24±234.54 | 1931.75±255.11 | 1890.13±177.14 |
| 7 | Glycine | 16120.47±2972.21 | 11474.94±2192.88 | 14303.56±2222.14 | 14207.90±2078.36 | 15437.80±1871.44 |
| 8 | Alanine | 31845.50±4179.61 | 39282.39±3985.84 | 36937.80±4312.97 | 33726.53±3899.24 | 31994.35±4287.02 |
| 9 | Hypoxanthine | 305.45±21.93 | 347.04±22.15 | 321.67±23.01 | 317.55±20.14 | 310.21±19.95 |
| 10 | Cytosine | 118.16±26.03 | 132.71±17.01 | 124.33±20.93 | 127.14±18.94 | 120.77±16.54 |
| 11 | Guanine | 108.26±14.93 | 89.97±11.77 | 101.23±12.51 | 97.33±14.10 | 107.58±10.75 |
| 12 | Adenosine | 125.18±19.67 | 106.95±15.90 | 126.13±14.32 | 123.18±20.45 | 144.76±17.78 |
| 13 | Uric Acid | 46191.60±4270.85 | 57042.62±5487.52 | 51237.44±4946.29 | 50003.25±4728.90 | 47271.39±5579.35 |
| 14 | Uracil | 111.70±26.82 | 151.09±15.50 | 121.47±20.78 | 119.98±22.55 | 117.56±13.72 |
| 15 | Carnitine | 6177.85±792.90 | 3891.54±728.14 | 5378.87±693.27 | 4097.72±598.31 | 4999.90±718.36 |
| 16 | Lactic Acid | 69191.76±7327.08 | 86938.15±7972.80 | 80923.56±7663.60 | 76893.48±7123.90 | 73110.25±7435.71 |
| 17 | S-Adenosy-L-Homocysteine | 23.79±5.42 | 27.33±4.97 | 13.97±2.89 | 22.29±3.42 | 14.07±3.82 |
| 18 | 5-Methyltetrahydrofolate | 140.81±22.69 | 151.10±23.85 | 80.65±21.61 | 130.81±20.09 | 87.61±18.06 |
| 19 | 5-Formyltetrahydrofolate | 6.66±0.91 | 5.96±0.89 | 1.78±0.93 | 5.51±0.78 | 1.71±0.99 |

Figure S1. (A) The ROC curves of the RA/Normal group, ROC curve-based model evaluation (AUC = 1.000). (B) Diagnostic efficacy evaluation using the ROC curves of the biomarkers between two groups. The optimal cutoffs using the closest to top-left corner and the area under ROC curves with a 95% confidence interval are displayed (AUC > 0.83).

**
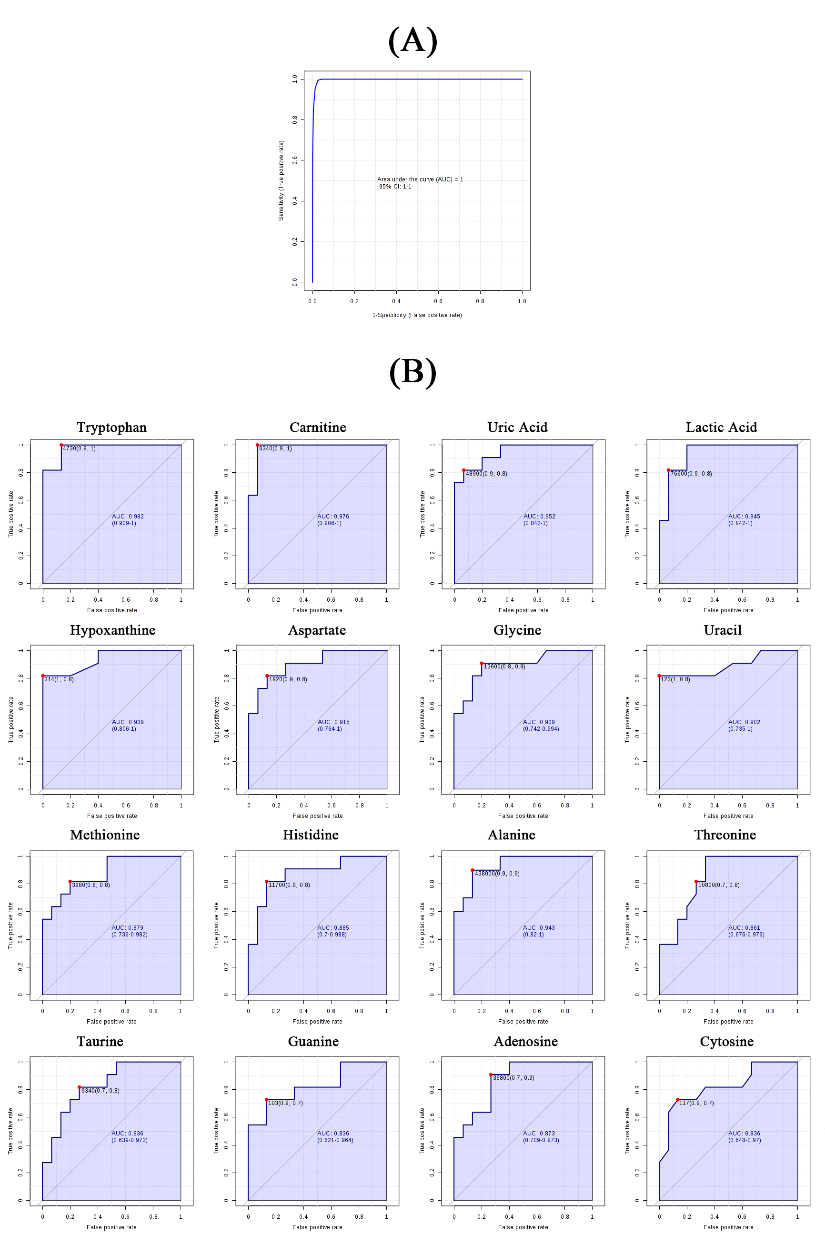
**
